# Compound‐Specific Stable Isotope Analysis Improves the Association Between Dairy Fatty Acid Biomarkers and Dairy Intake: A Secondary Analysis

**DOI:** 10.1002/lipd.70053

**Published:** 2026-04-01

**Authors:** Camilla Parzanini, Marisa Soo, Shirley Vien, Ji‐Eun Chon, Anthony J. Hanley, Priya Kathirvel, Bohdan Luhovyy, G. Harvey Anderson, Richard P. Bazinet

**Affiliations:** ^1^ Department of Nutritional Sciences University of Toronto Toronto Ontario Canada; ^2^ Mount Saint Vincent University Halifax Nova Scotia Canada

**Keywords:** δ^13^C, chronic diseases, dairy, isotopic composition

## Abstract

Increasing evidence suggests that dairy consumption may decrease the risk of chronic diseases. However, this association remains unclear due to methodological limitations. As a part of a secondary analysis, we used compound‐specific stable isotope analysis to increase the accuracy of the dairy FA biomarkers (15:0, 17:0), considering that each food item holds a unique stable C isotope (δ^13^C) signature. Healthy, overweight to obese adults (*n* = 96) were randomly assigned to one of 3 dietary treatments in a controlled multi‐site (Halifax and Toronto, Canada) clinical trial for 12 weeks: (i) calorie‐restricted diet; (ii) dairy‐rich diet, consisting of 1 serving each of full‐fat milk, cheese, and yogurt/day; and (iii) dairy‐rich + calorie‐restricted (DCR) diet. Content, composition, and δ^13^C signature of 15:0 and 17:0 were measured in the dairy foods provided, and participants' plasma and red blood cells. We found that the isotopic signatures of plasma 15:0 and 17:0 were more enriched in the high‐adherence group of participants in Halifax who consumed dairy (especially the DCR arm), and that these ratios were more similar to the signatures of the dairy food FA sources, thus reflecting dairy consumption. We also observed major differences in the content, composition, and isotopic signature of the target FA between the participants in Halifax and Toronto, which may be related to the different adherence rates, as well as to intrinsic geographic and dietary variations between populations. This study shows a potential use of compound‐specific stable isotope analysis to provide more accurate information on dairy FA biomarkers reflecting consumption.

**Abbreviations**
ANOVA: one‐way analysis of variance
BF3: boron trifluoride 14% in methanol solution
C‐DHQ II: Canadian diet history questionnaire II
CR: calorie‐restricted
D: dairy
DCR: dairy, calorie‐restricted
FA: fatty acid
FAME: fatty acid methyl esters
FID: flame ionization detector
GC: gas chromatography
IRMS: isotope ratio mass spectrometry
PERMANOVA: permutational analysis of variance
RBC: red blood cells
RCT: randomized controlled trial

## 1 Introduction

Chronic non‐communicable diseases, including cardiovascular diseases, type 2 diabetes, cancers, and chronic respiratory diseases, represent a global challenge and threat to the human population, as well as a serious burden to the public health system. According to the World Health Organization, 7 out of the 10 leading causes of death in 2021 were non‐communicable diseases, and these were responsible for the death of more than 43 million people that same year (WHO 2025), and this number expected to rise to 75.5 million deaths by 2050 (Freihat et al. 2025). Poor nutrition and dietary habits are among the main drivers of this epidemic (WHO 2025). It is hence critical to better understand the role of foods, including dairy products (e.g., milk, cheese, and yogurt), in chronic non‐communicable diseases. This will ultimately help guide national nutrition programs and dietary recommendations.

An increasing number of prospective observational studies, systematic reviews, and meta‐analyses report an inverse association between dairy food consumption and incidence of chronic diseases (e.g., Gil and Ortega 2019; cardiovascular diseases, Trieu et al. 2021, Key et al. 2019, de Oliveira Otto et al. 2013; type 2 diabetes, Bhavadharini et al. 2020, Imamura et al. 2018, Gijsbers et al. 2016). However, this relationship, as well as the underlying mechanisms, remain unclear. One of the factors contributing to discrepancies in the existing evidence relates to the methods used to assess dairy food intake. Food frequency questionnaires and dietary recalls are the most commonly utilized tools in nutritional epidemiologic studies, and allow measurement of intake of specific food items and nutrients in large populations at relatively low cost. However, food frequency questionnaires and dietary recalls are subject to intrinsic measurement errors and biases, and they may hence yield misclassified estimates of intake (Sellem et al. 2022; Yakoob et al. 2016; Van Dam and Hunter 2012). Furthermore, there is a lack of consistency in the food frequency questionnaires administered across studies.

To compensate for these limitations, more precise and objective techniques, including the analysis of fatty acid (FA) biomarkers, have been developed. For dairy foods, a series of FA have been identified as typical of these products and are commonly applied to assess dairy intake. Among these, the odd‐chain saturated pentadecanoic acid (15:0) and heptadecanoic acid (17:0) are the most frequently used FA biomarkers of dairy intake (Yakoob et al. 2016; Santaren et al. 2014; Brevik et al. 2005). These FA are produced via microbial fermentation in the rumen of cows and other ruminants (Sellem et al. 2022; Risérus and Marklund 2017) and have been associated with higher dairy consumption, as well as decreased risk of cardiovascular diseases (Trieu et al. 2021; de Oliveira Otto et al. 2013) and T2D (Santaren et al. 2019; Imamura et al. 2018; Mozaffarian et al. 2010) in prospective cohort studies. It is generally thought that humans are not able to synthesize these FA and, hence, their presence in human blood and adipose samples reflects dairy consumption (Wolk et al. 2001; Wolk et al. 1998).

However, there is concern regarding their exclusive use as biomarkers of dairy intake as, for instance, 15:0 and 17:0 can be found in foods other than dairy products, including ruminant meat and fish (Risérus and Marklund 2017; Ratnayake 2015). Ruminant meat (e.g., beef and sheep), in fact, presents 15:0 and 17:0 in similar proportions as in dairy fat, that is, 1.0% and 0.6%, respectively (Ratnayake 2015; Jensen and Newburg 1995); whereas fish contains on average 0.5% of 15:0 and 17:0 (Parzanini unpublished results) but these proportions may be far greater depending on the species, as in Capoccioni et al. (2018), for 15:0, and in Njinkoue et al. (2016), for 17:0. Furthermore, there is some evidence for endogenous synthesis of 15:0 and, particularly, 17:0 in humans (Venn‐Watson et al. 2020; Pfeuffer and Jaudszus 2016; Ratnayake 2015).

We therefore assessed the use of compound‐specific stable isotope analysis to overcome these limitations, a technique that combines the sensitivity of the capillary gas chromatography (GC) column with the high precision of isotope ratio mass spectrometry (IRMS). While stable isotope analysis is quite a common technique in geochemistry and, more recently, archeology and ecology studies, it is relatively new in the field of nutritional sciences (O'Brien 2015), especially when applied to specific compounds, such as FA (e.g., Smith et al. 2024; Klievik et al. 2023) vs bulk tissues. The rationale behind its use is that (i) primary producers (e.g., plants) are characterized by specific ^13^C to ^12^C ratios (δ^13^C) mainly due to their carbon fixation mode, that is, C_3_ vs C_4_ vs CAM plants (Troughton 1979; Bender 1968) and physiology (O'Leary 1981; Farquhar et al. 1982). Furthermore, (ii) the δ^13^C ratios of consumers reflect those of their food sources (McConnaughey and McRoy 1979; DeNiro and Epstein 1978).

With these considerations, the main objective of this exploratory secondary analysis was to assess the use of the isotopic signature of the dairy FA biomarkers, 15:0 and 17:0, by exploiting the natural variation in the relative abundance of the stable C isotopes (i.e., ^13^C to ^12^C) in participants in a controlled clinical trial exposed to dairy foods. In particular, we aimed to assess whether there were variations in blood levels and isotopic signatures of 15:0 and 17:0, as well as 16:0 (the most abundant FA present in dairy products), across dietary treatments and/or along with time. We hence hypothesized that (i) blood levels of 15:0 and 17:0 would be higher in participants consuming dairy products and would increase with the duration of consumption; and (ii) that the δ^13^C signatures of the dairy FA biomarkers (15:0, 17:0) in the blood of participants consuming dairy products would be more enriched and more similar to that of their dairy food sources.

## 2 Materials and Methods

### 2.1 Study Design

This investigation represents a secondary analysis of the primary RCT (trial ID# NCT04399460, https://www.clinicaltrials.gov/study/NCT04399460) that aimed to study the effects of consumption of full‐fat dairy on lean body mass, metabolic rate, and blood lipids. The full list of primary and secondary outcomes of the main investigation is provided in the link above. The original study (i.e., Anderson et al. 2026) was a single‐blinded, randomized, and parallel RCT conducted in two separate sites in Canada, i.e., Halifax and Toronto, under the lead of collaborators at Mount Saint Vincent University and University of Toronto, respectively. This study was conducted according to the guidelines laid down in the Declaration of Helsinki and approved by The University of Toronto Research Ethics Board and Mount Saint Vincent University Research Ethics Board before the commencement of the study at each site. Detailed information about the original study, including study design, participants, treatments, sample size, and full experimental protocol, is provided in Soo (2024).

Briefly, participants were recruited through study advertisements distributed as flyers, posters, postings around the cities of Halifax and Toronto, as well as local newspapers, websites, and social media. Eligibility was assessed via a screening questionnaire and, whenever eligible, participants were asked to provide written informed consent, as well as complete further questionnaires (i.e., Baseline Information Questionnaire, Eating Habits Questionnaire, and Diet History Questionnaire) and anthropometric measurements. Participants who satisfied all inclusion criteria were randomly assigned to one of three treatment groups in a ratio 1:1:1. Through the consent form, participants were made fully aware of any risks and discomfort that may have come from blood sampling procedures and eating packaged food provided to them (e.g., food‐borne illness); as well as that they may have felt some emotional discomfort while discussing body weight and/or during physical body measurements. Recruitment began in August 2020 and ended in June 2023. The full list of exclusion criteria is provided in Supporting Information: Appendix S1, while Figure S2 shows the flowchart of the six phases (i.e., pre‐screening, screening, enrollment, allocation, withdrawal, and analysis) of the original RCT.

The trial started in September 2020 and ended in January 2023, for the group of participants in Toronto, while it started in November 2021 and ended in September 2023, for the group in Halifax. Each trial was run for 12 weeks with biweekly follow‐up visits. The trial was paused in a few instances due to the COVID pandemic and shutdown of facilities, while the overall study ended upon the conclusion of the funding. Through a randomization schedule for a three‐treatment arm generated by SAS 9.4, the participants were randomly assigned to one of three dietary treatments using block randomization (block size = 12). Briefly, three random 3‐digit codes were generated for each site to conceal each arm of intervention. The code was assigned to each participant at enrolment in a sequential order following the parallel block randomization chart created before recruitment. A lead research coordinator at the University of Toronto generated the random allocation sequence, whereas trained postdoctoral fellows and/or graduate students enrolled and assigned participants to their specific intervention at each site. Participants were blinded to treatment allocation to avoid any effects on the outcome measurements.

The low dairy, calorie‐restricted (CR) treatment required a 500‐kcal energy restriction from the usual diet. The participants who underwent this treatment were also asked to reduce or maintain their low dairy intake and to use low‐fat dairy and/or plant‐based alternatives instead. The 3‐dairy + calorie‐restricted (DCR) treatment was also 500‐kcal restricted in an amount to accommodate the addition of 3 servings of full‐fat dairy per day, as follows: a serving (250 mL) of full‐fat milk for breakfast, a serving (100 g) of Greek yogurt prior to lunch, and a serving (42 g) of cheese prior to dinner. The yogurt and cheese options were interchangeable between lunch and dinner and had to be consumed 7–10 min prior to the meals. Last, the 3‐dairy (D) treatment required the participant to follow their usual diet while incorporating the 3 servings/day of dairy as described above. All treatment groups were encouraged to follow the 2019 Canadian Food Guide for dietary recommendations, with guidance provided by a registered dietician.

Dairy products were given to the participants every 2 weeks, and participants were asked to complete a dairy food log to monitor their dairy intake and assess adherence to the treatment. Products provided to the participants in Halifax and Toronto were from the same brand, except for milk as one brand was not available in the other province and *vice versa*. Nevertheless, the two milk products had the same fat content (3.25%) and similar nutritional values. Further details regarding the nutritional information of all the products are provided in Supporting Information: Appendix S6. Dairy samples (i.e., 10 mL of milk; *n* = 3–4 of 1 cm^2^ blocks of cheese; and 20 mL of yogurt) were collected prior to delivery to the participants, while blood samples were drawn every 4 weeks. Blood samples were centrifuged immediately after collection to allow for the separation of plasma and red blood cells (RBC). Dairy, and plasma and RBC samples were then stored in −20°C and −80°C, respectively, until further analysis. While plasma lipids (as total lipids and/or lipid pools) and RBC both reflect dietary fat intake, RBC are considered better predictors of long‐term intake (i.e., 3 weeks—3 months) due to their longer half‐lives (~27.8 days) and slower turnover rates (120 days) (Risé et al. 2007; Stanford et al. 1991). However, a large portion of the literature reports FA composition of plasma total lipids and/or lipid pools (Risé et al. 2007), especially when considering dairy FA biomarkers. For this reason, we included both blood components in this study.

### 2.2 Fatty Acid Extraction, Methylation, and Analysis

Total lipids and FA were extracted using a modified version of Folch et al. (1957) and analyzed as methyl esters (FAME). Specifically, known aliquots of dairy and plasma samples (milk, ∼200 mg; yogurt, ∼1000 mg; cheese, ∼50 mg; plasma, 100–200 μL) were weighed and transferred into clean test tubes. A 1.75 mL aliquot of 88% KCl was then added to each sample, followed by 2 mL of methanol, 4 mL of chloroform, and a known amount of the internal standard nonadecanoic acid,19:0 (50 μg for dairy products, 40 μg for plasma samples). Samples were vortexed and then centrifuged for 10 min at 1460 rpm to induce the separation of two layers. The bottom layer, containing the chloroform phase and lipid extract, was pipetted out into new test tubes and lipid extracts were dried down under a gentle stream of N_2_. Once evaporated, 1 mL of hexane and 1 mL of a boron trifluoride 14% in methanol solution (BF_3_) were added to the dry samples and the tubes tightly capped, gently vortexed, and then incubated in a pre‐heated oven at 100°C for 1 h. Once out from the oven, the samples were let to cool down for 5 min, and then 2 mL of milliQ water were added to stop the methylation reaction from occurring. Samples were vortexed and then centrifuged for 10 min at 1460 rpm to allow for the separation. The top layers containing the FAME in hexane were added into chromatography vials and dried down under N_2_. Dairy was reconstituted in 0.5–2 mL hexane, while the plasma was reconstituted in 100–200 μL hexane.

As for the RBC extraction, an aliquot of ∼25 mg was weighed and transferred into test tubes. An aliquot of 1 mL of BF_3_ and 0.3 mL of hexane containing 5 μg of the internal standard 19:0 was added to the tubes, which were then capped and vortexed. Samples were placed on a heat block at 95°C for 1 h and left to cool down for 10 min before adding 1 mL hexane and 1 mL of water. Samples were then vortexed, centrifuged at 1460 rpm for 5 min, and the upper hexane layer with the FAME extracts was transferred into chromatography vials. Last, FAME extracts were dried down under N_2_ and reconstituted in 75 μL heptane.

The FAME extracts were analyzed by GC, using a Varian 430 GC equipped with an auto‐sampler and a flame ionization detector (FID). The column DB‐FFAP (30 m × 0.25 mm × 0.25 μm; Agilent, 122‐3232) was used. The column oven program was initially set at 50°C and held for 1 min. The column temperature then ramped to 130°C at a rate of 30°C/min, to 175°C at 10°C/min, to 230°C at 5°C/min and held for 9.5 min and, lastly raised to 240°C at a rate of 50°C/min. The final temperature was held for 11.13 min, for a total of 40 min. Peaks were identified by comparing retention times from known standards, including a GLC‐569 FAME mix (Nu‐Check Prep Inc., Elysian, MI, catalogue # GLC‐569), a dairy FA mix made in the lab using individual FAME standards, including 15:0 and 17:0 (Nu‐Check Prep Inc., Elysian, MI, catalogue # N‐15‐M and N‐17‐M, respectively). In this study, FAME data were reported as molar concentrations (i.e., for dairy product and RBC samples, μmol/g or nmol/g; for plasma samples, μmol/L) and molar percentages (mol%) of total identified FAME, that is, as relative proportions of individual molar FAME amounts.

### 2.3 Compound‐Specific Stable Isotope Analysis

The δ^13^C ratios of FAME 15:0, 16:0, and 17:0 in dairy products, plasma, and RBC were measured via GC‐IRMS; Thermo Scientific Trace 1310 GC; MAT 253 IRMS, Thermo Finnigan MAT, Bremen, Germany. A TriPlus RSH autosampler (Thermo Fisher Scientific) injected aliquots (1–5 μL) of samples, together with the dairy FA mix described above, into a capillary column (100 m, 0.25 mm i.d., 0.20 μm df; Supelco SP‐2560; Sigma‐Aldrich) through a GC Iso Link II combustion interface (Thermo Scientific). The temperature was initially set at 60°C and then raised to 180°C at 15°C/min with no hold, and to 240°C at 1.5°C/min with an 18 min hold, leading to a total run time of 66 min. The carrier flow rate was set to 1.2 mL/min, yielding baseline resolutions of analyte peaks of interest. With helium as the carrier gas, the GC effluent was swept to a Thermo Fisher Scientific GC Iso Link II combustion interface at 1000°C, with nickel and copper catalysts, and the MAT 253 IRMS (Thermo Fisher Scientific) was interfaced via a ConFlo IV (Thermo Fisher Scientific) continuous‐flow interface. Prior to entering the IRMS ion source (Dupont, Wilmington, DE), the CO_2_ gas produced by quantitative combustion of isolated analytes was dried by flowing the gas through a Nafion dryer.

**FIGURE 1:**
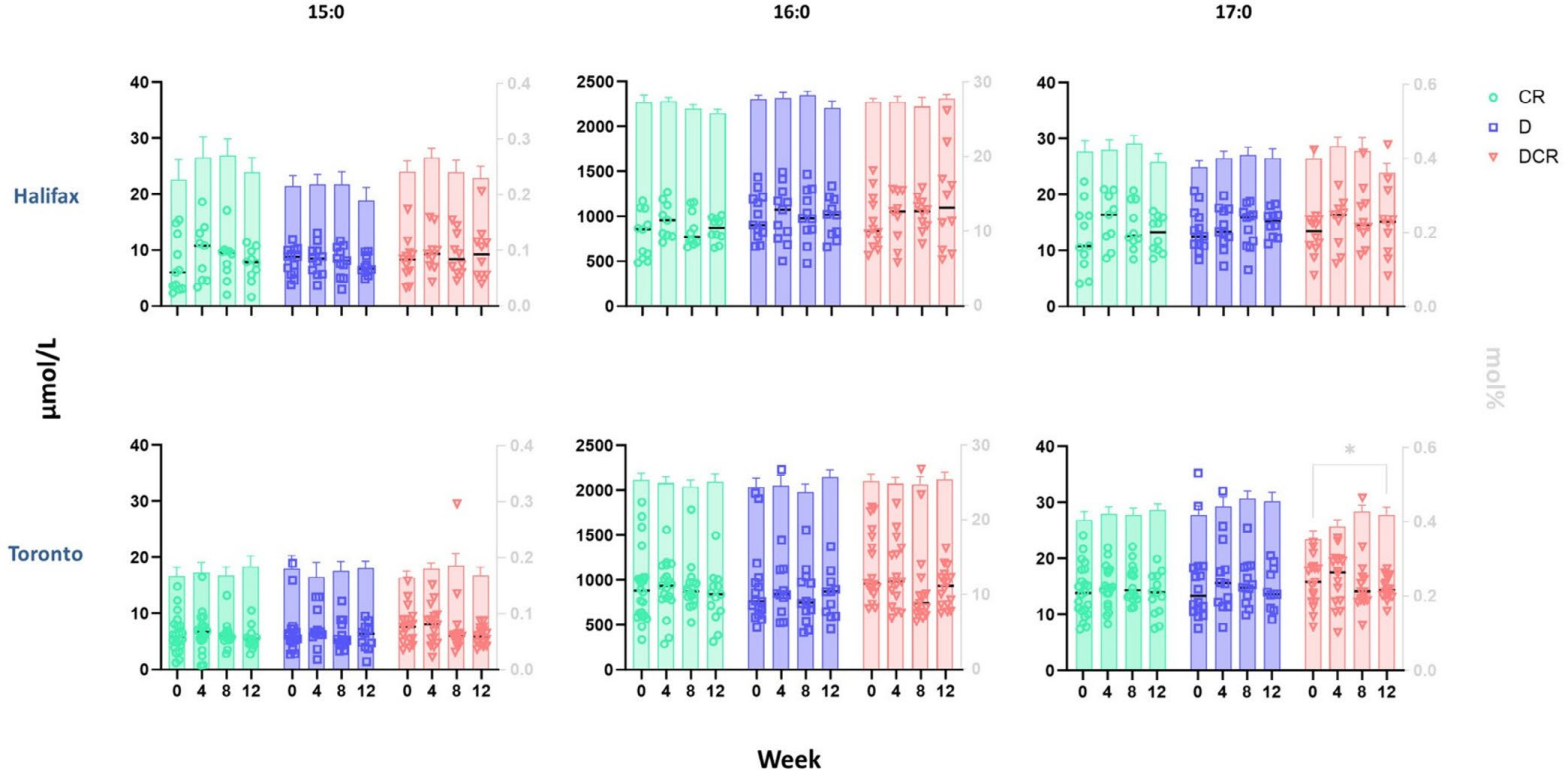
Levels of the dairy fatty acids (i.e., 15:0, 16:0, and 17:0) measured in the plasma of the participants from Halifax and Toronto, across the calorie restricted (CR), dairy (D), and dairy‐calorie restricted (DCR) treatments, and at weeks 0, 4, 8, and 12. Individual symbols represent the molar amounts (μmol/L) of the fatty acid biomarkers measured in the participants and refer to the left *Y* axis, while bars represent the molar percentages (mol%) and refer to the right *Y* axis. Moreover, green symbols and bars illustrate the values of the fatty acid biomarkers in participants who underwent the CR treatment. Blue symbols and bars indicate the values measured in the participants who ate dairy (D), and pink symbols and bars represent the values measured in the participants who were assigned to the DCR treatment. Black horizontal lines report the median FA amounts, while asterisks highlight significant (*p* < 0.05) Tukey's pairwise comparisons, following mixed‐effects analysis, and error bars represent standard errors (Halifax, n_CR_ = 40, n_D_ = 45, n_DCR_ = 41; Toronto, n_CR_= 77, n_D_ = 52, n_DCR_ = 64; across weeks).

**FIGURE 2:**
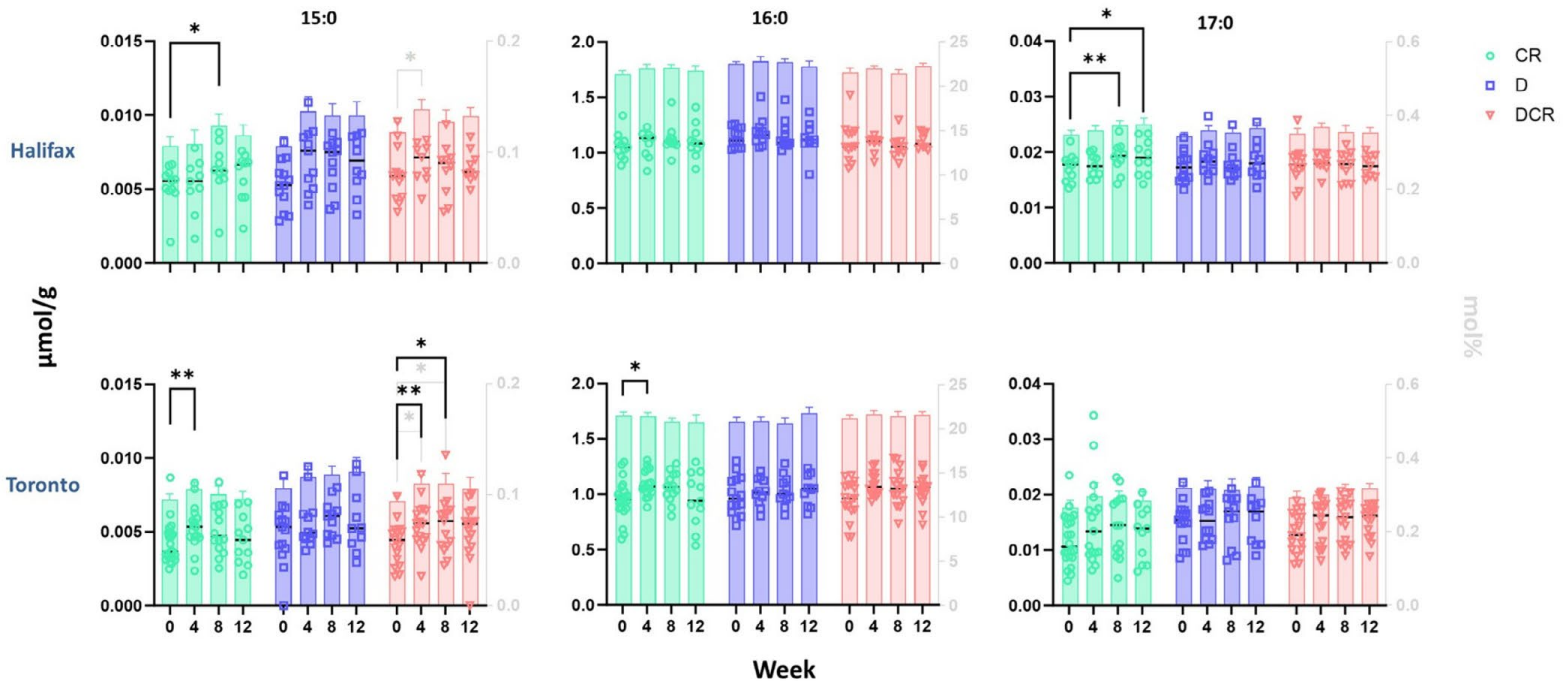
Levels of the dairy fatty acids (i.e., 15:0, 16:0, and 17:0) measured in the red blood cells RBC of the participants from Halifax and Toronto, across the calorie restricted (CR), dairy (D), and dairy‐calorie restricted (DCR) treatments, and at weeks 0, 4, 8, and 12. Individual symbols represent the molar amounts (μmol/g) of the fatty acid biomarkers measured in the participants and refer to the left Y axis, while bars represent the molar percentages (mol%) and refer to the right *Y* axis. Moreover, green symbols and bars illustrate the values of the fatty acid biomarkers in participants who underwent the CR treatment. Blue symbols and bars indicate the values measured in the participants who ate dairy (D), and pink symbols and bars represent the values measured in the participants who were assigned to the DCR treatment. Black horizontal lines report the median FA amounts, while asterisks highlight significant (*p* < 0.05) Tukey's pairwise comparisons, following mixed‐effects analysis, and error bars represent standard errors (Halifax, n_CR_ = 40, n_D_ = 45, n_DCR_ = 40, Toronto, n_CR_ = 64, n_D_ = 48, n_DCR_ = 63; across weeks).

After running several samples via GC‐IRMS, we noticed that the 15:0 and 17:0 peaks in RBC samples were below the detection limit. To avoid obtaining imprecise isotopic values, we hence decided not to continue the analysis of RBC samples and to only consider the isotopic results from dairy products and plasma samples in this investigation. This issue could have been due either to a technical problem with the equipment and its column or to insufficient RBC mass used during lipid extraction. Overall, the relative amounts of 15:0 and 17:0 were relatively low compared to other FA present in the participants' plasma and RBC samples (Figures 1 and 2).

Furthermore, a random subsample of dairy products was analyzed (*n* = 9 per sampling site). The δ^13^C isotope ratios were reported in milliUrey (mUr; 1 mUr = 1‰) and normalized using consensus‐validated 20‐carbon FAME reference material USGS70, USGS71, and USGS72 (Reston Stable Isotope Laboratory). The consensus‐derived carbon isotope ratios of USGS70, USGS71 and USGS72 measure −30.53, −10.50, and −1.54 mUr, respectively, and are expressed relative to the international reference standard for carbon isotopes, that is, Vienna Peedee Belemnite, on a scale normalized to primary reference materials NBS 19 and LSVE (Schimmelmann et al. 2016).

### 2.4 Diet History Questionnaire Calculations

The Diet History Questionnaire provided was a modified version of the Past‐Month Canadian Diet History Questionnaire II (C‐DHQ II) from the U.S. National Cancer Institute (https://epi.grants.cancer.gov/dhq2/). The C‐DHQ II was provided prior to the start of the intervention, as well as at the end of the trial. Modifications were made to include questions about dairy milk alternatives (e.g., almond and coconut milk). While these questions are present in the version III of the DHQ for U.S., the C‐DHQ III counterpart has not been released yet. Due to these changes made to the DHQ, it was not possible to obtain the complete nutrient and food group intake estimates from the participants using the Diet*Calc software available on the website. Nonetheless, we calculated the consumption, over the past month, of those food items that are known to include the target FA, in addition to cheese, milk, and yogurt, along with other food items that may affect the bulk δ^13^C signature (Bataille et al. 2020). This analysis provided us with a better depiction of the participants' dietary habits and complemented the main biomarker assessment. Specifically, we used frequency information (e.g., “How often in the past month…”) and quantities of foods (e.g., cups and ounces), converted into “servings” based upon the participants' responses, as well as the nutrition labels of Canadian or North American products, to estimate consumption rates as serving/day. Results of this analysis are provided as Figures S3 and S4, whereas further explanation on the calculations may be found in Supporting Information: Appendix S5.

### 2.5 Statistical Analysis

Although the energy restriction component of the trial was not relevant in this secondary analysis, we decided to keep the three‐treatment arm structure to control for potential variability in the diet across treatments. A series of permutational analysis of variance (PERMANOVA) models were run to test the effect of sampling site (Halifax, Toronto) and treatment on plasma and RBC content and composition, as well as plasma isotopic composition of 15:0, 16:0, and 17:0. PERMANOVA is a multivariate statistical test which is commonly used to analyze FA data—especially in the ecology field (e.g., Galloway et al. 2012)—, as it allows to test (i) the effect of a variety of predictors (e.g., sampling site, treatment) on the FA composition; and, (ii) if there are any differences among different groups or samples in terms of FA composition. This technique is especially useful when analyzing complex datasets as is the case in our study. Considering that the PERMANOVA tests highlighted major significant differences (p(perm) ≤ 0.01) between sampling sites (Halifax vs Toronto; Table S7), and a significant Sampling site × Treatment interaction for the isotopic measurements, subsequent univariate statistical tests were performed considering the samples from the two sites separately.

A series of unpaired t (t) or Mann–Whitney (U; non‐parametric) tests were run to assess the differences in the FA content and composition, as well as isotopic composition of 15:0, 16:0, and 17:0 measured in the cheese, milk, and yogurt samples provided to the participants in Halifax vs Toronto. T‐statistics are here reported with their degrees of freedom, as *t*_df_. Moreover, mixed‐effects analysis coupled with Tukey's multiple comparison test were used to explore differences in the FA content and composition, as well as in the isotopic composition of the target FA across treatments (i.e., CR, D, and DCR) and along with time (i.e., weeks 0, 4, 8, and 12). Last, one‐way analysis of variance (ANOVA) was run to further investigate differences in the isotopic composition of plasma 15:0, 16:0, and 17:0 across treatments. Noting that this study is a secondary analysis, the sample size was initially calculated using a standard deviation (SD) = 3.93 kg, with estimated power = 0.80, and *α* = 0.05 to detect significant changes in body weight following dairy consumption. Statistical significance was here set at *p* < 0.05. Multivariate statistics were performed using Primer 7.0, with the PERMANOVA+ add‐on package, whereas univariate statistics was conducted using GraphPad Prism 9.

## 3 Results

### 3.1 Participants Baseline Characteristics

The FA data included in this analysis were derived from *n* = 96 healthy, overweight to obese (body mass index, 24.6–42.5 kg/m^2^), male and female adults (23–60 years old), with no dietary allergies. Table 1 reports further details on the baseline characteristics of these participants according to the sampling location, including weight, height, waist circumference, and ethnicity information.

**TABLE 1:** Baseline characteristics of the participants in Halifax and Toronto included in this study.

Variables	Values			
	*n*	%	Mean	SD
Halifax				
Number of participants^a^	36	37.5		
Sex: Female	20	55.6		
Age (years)			41.4	9.8
Weight (kg)			85.7	11.8
Height (cm)			171.1	10.2
BMI^b^ (kg/m^2^)			29.3	3.0
Waist circumference (cm)			101.5	10.3
Ethnicity				
East Asian	1	2.8		
Not or mis‐recorded	25	69.4		
South Asian	3	8.3		
White	7	19.4		
Toronto				
Number of participants^a^	60	62.5		
Sex: Female	31	51.7		
Age (years)			33.3	6.9
Weight (kg)			87.1	18.4
Height (cm)			170.4	10.6
BMI^b^ (kg/m^2^)			30.0	4.1
Waist circumference (cm)			100.0	11.9
Ethnicity				
African	1	1.7		
Afro‐Caribbean	1	1.7		
East Asian	9	15.0		
Latino	4	6.7		
Not or mis‐recorded	22	36.7		
South Asian	9	15.0		
Southeast Asian	2	3.3		
West Asian descent	2	3.3		
White	10	16.7		

^a^ The percentage value represents the fraction of participants in each sampling site in relation to the total number of participants in Halifax + Toronto.

^b^ BMI, body mass index.

### 3.2 Adherence to the Dairy Treatment

A total of 21 participants in Halifax who were randomized to consume dairy items filled in the dairy food log; however, only 17 of these participants did so for the whole duration of the trial. When considering the dairy‐food‐log data from all the participants, the mean adherence rate was 87% ± 12%, while this percentage increased to 88% ± 10% when considering only the data of those who filled in the dairy food logs for the whole trial period (Table S8). Overall, the median value of adherence of the population of participants in Halifax was 89%.

In contrast, 32 participants in Toronto who were randomized to consume dairy products filled in the dairy food logs, but only 28 of them completed it over the whole 12‐week trial (Table S9). Considering the data of all the 32 participants who filled in some records, the adherence to the dairy treatment assigned was 69% ± 26%, while this proportion increased to 76% ± 22% when including only data of the participants who completed the log over the 12‐week trial. The median value for the Toronto population was 79%.

### 3.3 Variations in Fatty Acid Content and Composition of Dairy Products, Plasma, and RBC

Significant differences were detected in the FA contents of the dairy products provided to the participants in Halifax vs Toronto (Table 2). In particular, the cheese samples provided to the participants in Halifax had lower amounts of 15:0 (*t*_72_ = 3.957, *p* < 0.001), 16:0 (*t*_72_ = 3.376, *p* < 0.010), and 17:0 (*t*_72_ = 3.441, *p* = 0.001). Similarly, the milk and yogurt samples provided to the participants in Halifax were characterized by lower amounts of 17:0 (*U* = 143, *p* < 0.010), 15:0 (*U* = 2126, *p* < 0.010), and 16:0 (U = 2364, *p* = 0.03), respectively, compared to the amounts measured in the dairy samples given to the participants in Toronto.

**TABLE 2:** Mean amounts (μmol/g) and molar percentages (mol%) ± standard deviation (SD) of 15:0, 16:0, and 17:0 measured in the dairy products (i.e., cheese, milk, and yogurt) provided to the participants in Halifax and Toronto.

Dairy Type	Sampling Site	*n*	15:0			16:0			17:0			15:0			16:0			17:0		
			μmol/g			μmol/g			μmol/g			mol%			mol%			mol%		
			Mean	SD		Mean	SD		Mean	SD		Mean	SD		Mean	SD		Mean	SD	
Cheese	Halifax	38	0.84	0.24	a	26.54	7.17	a	0.44	0.11	a	1.21	0.11	A	38.08	2.71		0.64	0.05	
	Toronto	36	1.12	0.36	b	33.20	9.67	b	0.56	0.17	b	1.28	0.14	B	37.95	2.73		0.64	0.07	
Milk	Halifax	40	0.12	0.05		3.49	1.60		0.05	0.02	a	1.25	0.13	A	37.28	2.23		0.55	0.09	A
	Toronto	15	0.14	0.05		3.82	1.14		0.08	0.03	b	1.38	0.20	B	36.92	1.07		0.72	0.19	B
Yogurt	Halifax	87	0.05	0.03	a	1.63	0.80	a	0.03	0.01		1.14	0.17	A	35.31	2.45	A	0.66	0.11	A
	Toronto	68	0.06	0.02	b	1.76	0.55	b	0.03	0.01		1.31	0.14	B	36.59	1.80	B	0.64	0.09	B

*Note:* Samples sizes (*n*) are also provided. Lower‐case (i.e., a, b) and upper‐case letters (i.e., A, B) report significant differences (t‐ and Mann–Whitney tests, *p* < 0.05) in the fatty acid biomarker molar amounts and percentages, respectively, measured in the dairy products provided to the participants in Halifax vs Toronto.

Similarly, significant differences were also observed when comparing the molar percentages (mol%) values (Table 2). Specifically, mol% of 15:0 in the dairy products provided to the participants in Halifax were lower than those in the dairy products provided to the participants in Toronto (cheese, *t*_(72)_ = 2.457, *p* = 0.02; milk, *t*_(53)_ = 2.887, *p* = 0.006; yogurt, *U* = 1369, *p* < 0.0001). Moreover, yogurt 16:0 (*U* = 1915, *p* < 0.001) and 17:0 mol% (*U* = 2203, *p* = 0.006), as well as milk 17:0 mol% (*U* = 79, p < 0.0001), were lower in the dairy products provided to the participants in Halifax.

As mentioned above, multivariate statistics highlighted significant differences between sampling sites (Halifax vs Toronto; p(perm) ≤ 0.01) in the molar amounts, molar percentages, and isotopic compositions of the target FA (Table S7). For this reason, further univariate statistical tests were performed considering the samples from the two sites separately. Specifically, mixed‐effects analysis revealed no major trends among treatments (i.e., CR, D, and DCR) nor across time (i.e., 0, 4, 8, and 12 weeks) nor any interaction effect of treatment and time on the amounts and molar percentages of 15:0, 16:0, and 17:0 measured in the plasma and RBC samples of the participants in Halifax and Toronto. Nevertheless, there were a few pairwise differences between weeks, when comparing values within treatments, which are shown in Figures 1 and 2. Table S10 presents the 15:0, 16:0, and 17:0 molar amounts and percentages characterizing the plasma of the participants in Halifax and Toronto, while those measured in the RBC samples are provided in Table S11.

### 3.4 Variations in the Fatty Acid Isotopic Signature of Dairy Products and Plasma

**FIGURE 3:**
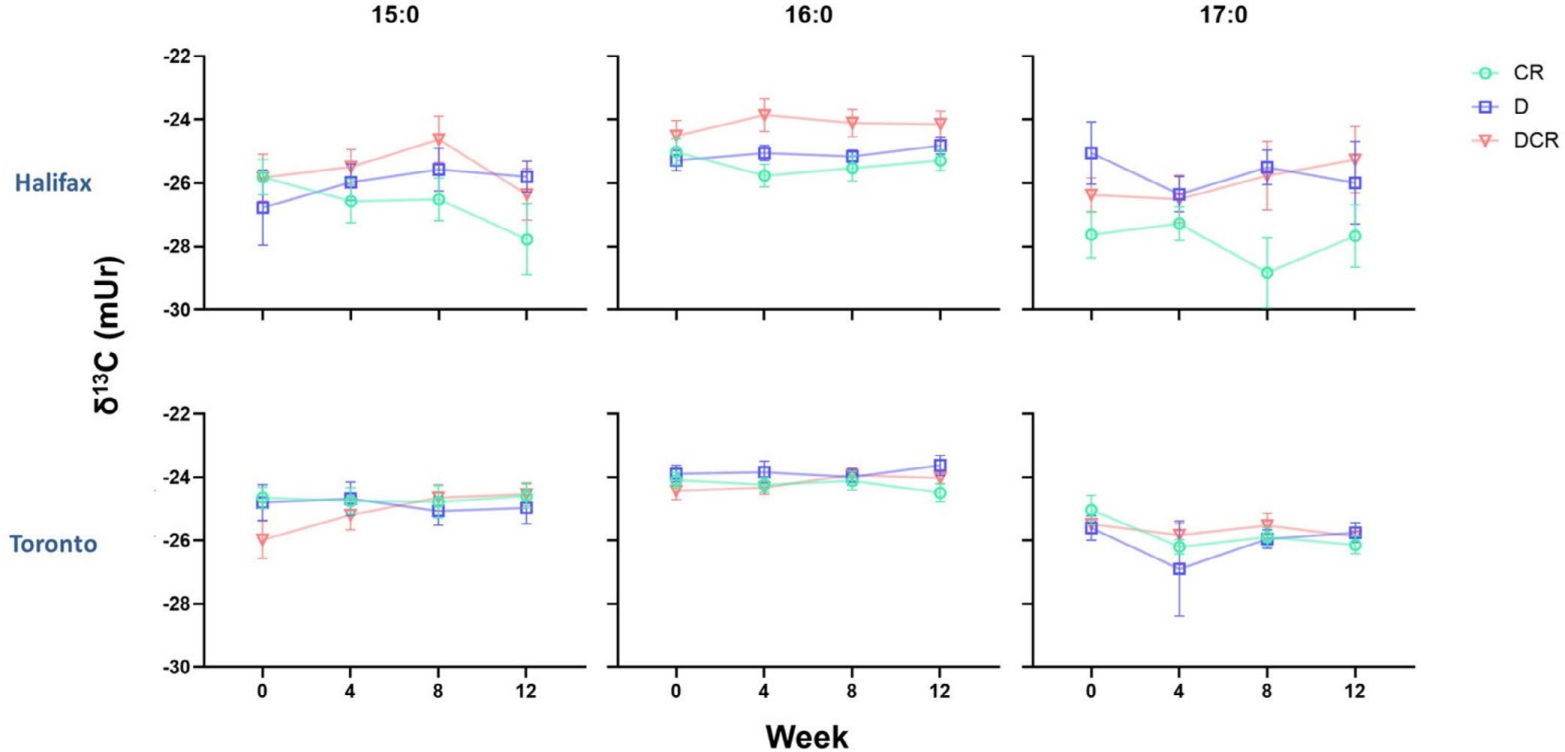
Mean δ^13^C ratios of 15:0, 16:0, and 17:0 measured in the plasma of the participants in Halifax and Toronto, across the calorie restricted (CR), dairy (D), and dairy‐calorie restricted (DCR) treatments, and at week 0, 4, 8, and 12. Specifically, green symbols and bars refer to the δ^13^C ratios of the fatty acid biomarkers measured in the participants who underwent the CR treatment. Blue symbols and bars represent the δ^13^C ratios measured in the participants who ate dairy (D), and pink symbols and bars represent the δ^13^C ratios measured in the participants who were assigned to the DCR treatment. Error bars represent standard errors (Halifax, n_CR_ = 38, n_D_ = 40, n_DCR_ = 37; Toronto, n_CR_ = 62, n_D_ = 45, n_DCR_ = 66; across weeks and per each fatty acid).

**FIGURE 4:**
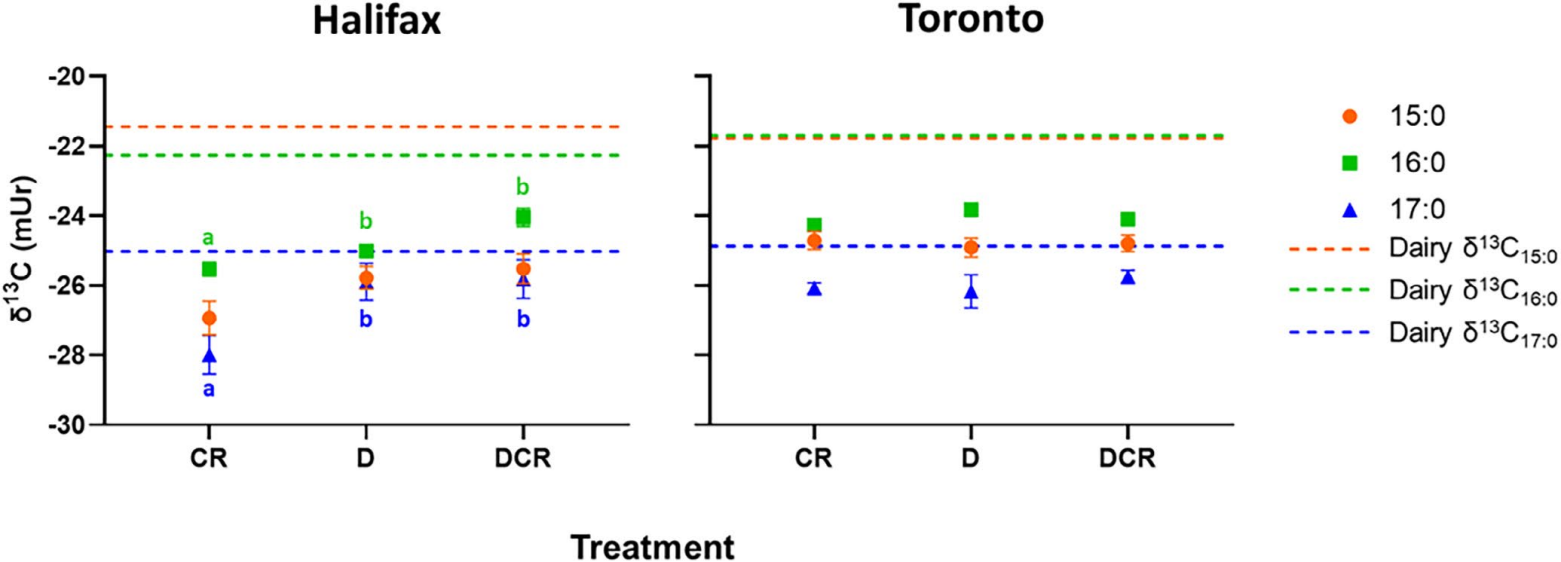
Mean δ^13^C ratios of 15:0 (orange circles), 16:0 (green squares), and 17:0 (blue triangles) measured in the plasma of the participants in Halifax and Toronto, across the calorie restricted (CR), dairy (D), and dairy‐calorie restricted (DCR) treatments. Samples at weeks 4, 8, and 12 were grouped for each treatment. Error bars represent standard errors (Halifax, n = 24–32; Toronto, n = 33–47; across treatements and fatty acids), while a letter code highlights significant (*p* < 0.05) Tukey's pairwise comparisons across treatments following one‐way analysis of variance (Table S13), and dashed lines identify the δ^13^C ratios of 15:0 (orange), 16:0 (green), and 17:0 (blue) measured in the dairy food items provided to the participants.

No differences were found in the δ^13^C ratios of 15:0, 16:0 and 17:0 measured in the dairy products given to the participants in Halifax vs Toronto (Figure S12), nor in the δ^13^C ratios of plasma 15:0, 16:0 and 17:0 across time (Figure 3). Nonetheless, mixed effect analysis highlighted significant variations across treatments for the participants in Halifax. Specifically, the δ^13^C ratios of plasma 16:0 and 17:0 in the treated participants in Halifax (weeks 4, 8, and 12 pooled) who consumed dairy were more enriched compared to those measured in the participants not exposed to the dairy treatment (ANOVA, *p* < 0.01; specific test results are reported in Table S13). Not only were these ratios more enriched, but they were also more similar to the ratios of their dairy food sources (Figure 4).

**FIGURE 5:**
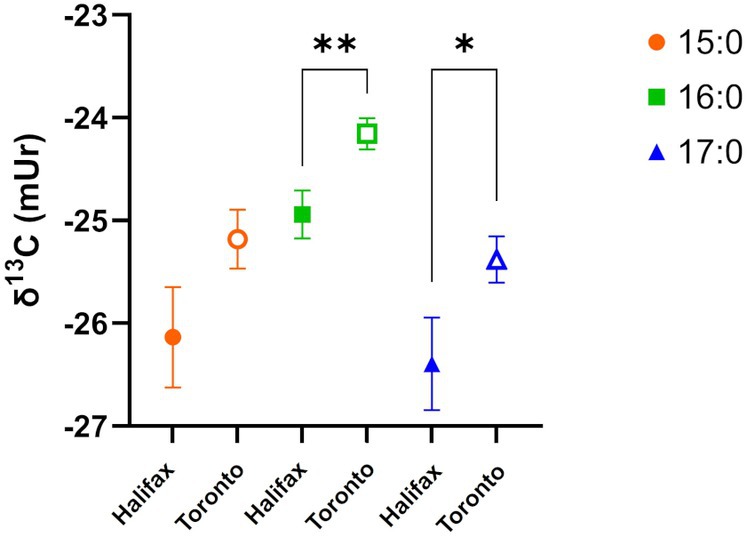
Mean δ^13^C ratios of 15:0 (orange circles), 16:0 (green squares), and 17:0 (blue triangles) measured in the plasma of the participants in Halifax (filled symbols) and Toronto (empty symbols) at baseline (week 0). Error bars represent standard errors (Halifax, n_15:0_ = 30, n_16:0_ = 33, n_17:0_ = 28, Toronto, n_15:0_ = 47, n_16:0_ = 48, n_17:0_ = 48), while asterisks indicate significant differences (*p < 0.05, **p < 0.01) between sampling sites revealed by unpaired *t*‐tests.

In contrast, no trend was observed in the δ^13^C ratios measured in the plasma of the participants in Toronto. Interestingly, the δ^13^C ratios of the target FA measured in the participants in Halifax at baseline (week 0) were more depleted compared to those quantified in the plasma of the participants in Toronto (δ^13^C_16:0_, *t*_79_ = 2.949, *p* < 0.01; δ^13^C_17:0_, *t* = 2.247, *p* = 0.03) (Figure 5). Table S14 reports all the δ^13^C ratios of 15:0, 16:0, and 17:0 according to sampling team, treatment, and week.

These results were reinforced when comparing low‐ vs high‐adherence groups of participants in Halifax and Toronto, based on their relative median values (89% and 79%, respectively). In particular, the high‐adherence group of participants in Halifax consuming dairy foods exhibited more enriched δ^13^C ratios of the target FA compared to those who did not eat dairy products (Figure S15, Table S16).

## 4 Discussion

### 4.1 Isotopic Differences Between Halifax vs Toronto Participants

The δ^13^C ratios of the dairy FA biomarkers 15:0 and 17:0 in the plasma samples of the high‐adherence group of subjects in Halifax were more enriched in participants consuming dairy products (especially the DCR arm) and more similar to those of the dairy products provided, thus reflecting dairy consumption for this population of participants. In contrast, no differences were observed in the participants in Toronto, despite the Past‐Month C‐DHQ II data showing an increased consumption of dairy products at week 12 in participants who were randomized to eat cheese, milk, and yogurt daily as per instructions, in both Halifax and Toronto (Figure S4). These results, which partly confirmed one of our initial hypotheses, could be due to several reasons, including participants' adherence. In this regard, adherence to the dairy treatments (i.e., D and DCR) was in general higher for the participants in Halifax (89% vs 79%). While 79% of adherence is still considered relatively “high,” it was not as high as for the participants in Halifax, and in fact only *n* = 4 participants in Toronto out of 32 presented rates > 89% (Table S9).

Assuming that, overall, the information provided through the dairy‐food log was accurate and represented the actual participants' behaviors, the participants in Toronto consumed relatively fewer servings of the dairy products provided to them, thus decreasing the effectiveness of this analysis for this population, even when low‐ vs high‐adherence groups were analyzed separately. Additionally, the isotopic data were already different at baseline (week 0) (i.e., generally, more depleted δ^13^C ratios of the target FA in the plasma of Halifax participants), thus providing another possible explanation for the lack of response for the participants in Toronto. In other words, the lack of a major difference in the isotopic composition at baseline between the dairy foods provided and the Toronto participants most likely masked any effects of the dairy treatment throughout the trial. For this reason, the two populations should be considered as separate entities, as also indicated by multivariate statistics performed on the isotopic data, as well as on the FA molar content and composition. This difference at baseline should be independent of the dairy food sources provided to the participants, given the similar δ^13^C ratios between the Halifax and Toronto dairy products, and it may be instead attributed to intrinsic geographic differences between the two populations, as well as to inter‐individual dietary variations.

Geographic variations in the δ^13^C signatures of Canadians from different parts of the country have already been observed previously by Bataille et al. (2020) and mostly ascribed to differences in the major dietary sources and, indirectly, environmental conditions that drive the type and amounts of plant species across regions. Specifically, Bataille and coauthors measured the bulk stable C isotope composition of hair samples collected from participants across Canada to gain further insights into their nutrition, and they found that the δ^13^C ratios reflected the major type of crop(s) grown within each specific province. Corn, which is a C_4_ plant, is a source of feed for cattle in Ontario, Quebec, and Eastern Canada overall. This leads to more enriched δ^13^C ratios in meat and dairy products from these areas, as well as in consumers of these foods, compared to the Western Canada regions which mostly rely on wheat and barley. Wheat and barley are C_3_ plants, which are characterized by more depleted δ^13^C signatures compared to C_4_ plants (bulk mean ratios, C_3_ plants, −27.80 mUr; C_4_ plants, ~ −13.56 mUr; Troughton 1979). Similar conclusions using human hair and fingernail samples, although pertaining to different geographical regions, were also obtained by Ammer et al. (2020), within Mexico; Hülsemann et al. (2015), who looked at variations at the global scale; and Nardoto et al. (2006) who compared western US individuals with Brazilian individuals living in urban areas and in a moderately isolated area in the central Amazon region.

Concerning the isotopic signature of the dairy food sources provided to the participants in this study, our results are in line with what has been reported by Bataille et al. (2020). Specifically, the dairy food products bought in Halifax and Toronto exhibited similar isotopic signatures, suggesting similar agricultural production practices. Moreover, considering the mean bulk δ^13^C ratios of C_3_ vs C_4_ plants reported above, the signatures of the dairy food products analyzed in this study were more enriched in ^13^C, indicating that cattle from Ontario and Nova Scotia consume at least some corn. Our ratios are also similar to previous measurements obtained in our laboratory with samples of dairy items (i.e., cheese, milk, and yogurt) purchased from various stores in Ontario and human serum samples, all collected as part of a study by Chouinard‐Watkins et al. (2021) and analyzed separately (unpublished results). Specifically, it was found that the δ^13^C ratios of the major FA present in dairy products, including 16:0, were generally more enriched (i.e., about −20 mUr) compared to ratios of the same FA measured from the subjects' serum (i.e., −30 to −24 mUr).

Based on Bataille et al. (2020), one might also expect, at first, more comparable isotopic signatures between the two populations of participants, given that they most likely rely upon similar agri‐food systems for food consumption. However, while the Past‐Month C‐DHQ‐II data showed little difference in the consumption of relevant food items between populations at baseline (Figure S3), thus suggesting similar dietary habits between the two populations; the FA levels and their δ^13^C ratios measured in the participants' plasma were different, which pointed to opposite interpretations. Considering that (i) the dietary information we collected from the C‐DHQ‐II only partially captured the participants' dietary habits; that (ii) we do not know the actual origin of the dietary items included in the analysis; and that (iii) we did not measure the isotopic composition of the participants' food sources, we cannot fully compare the C‐DHQ‐II data with the isotopic results in this study.

Furthermore, given that the δ^13^C ratios of plasma 15:0 and 17:0 should be the result of a mixture of δ^13^C ratios from all foods containing those specific FA (e.g., meat and fish) and that, as mentioned before, we did not measure the isotopic composition of these other food items, we still cannot completely exclude that the two populations were characterized by different diets, at least in qualitative terms, with diversified intakes of dairy products and other food items containing 15:0 and 17:0, as the isotopic data indicate. Variations in age, sex, ethnicity, and social status, may also add to the difference in the isotopic data between populations, as well as among individuals, as shown in previous studies using historical tissue samples (Reitsema and Vercellotti 2012; Barrett et al. 2001; Katzenberg 1993). However, these factors were not here considered as they were outside the scope of this investigation.

### 4.2 Variations in FA Content and Composition of Dairy Products and Blood Samples

Whereas the isotopic signatures of 15:0, 16:0, and 17:0 measured in dairy products provided to the participants in Halifax vs Toronto were similar (although we analyzed a small random subsample), a few differences were observed when looking at their FA contents. Overall, the dairy foods provided to the participants in Toronto tended to have higher contents of the target FA, with a few exceptions. Nevertheless, this difference may be considered negligible for 15:0 and 17:0 which were present at low mean amounts and relative molar proportions (≤ 1.12 μmol/g and ≤ 1.38 mol% for 15:0; ≤ 0.56 μmol/g and ≤ 0.72 mol% for 17:0).

Regardless, a certain spatial and seasonal variation in the FA content and composition of dairy foods, even when produced within the same plant, is to be expected. Year and season are the main determinants of milk FA composition in a study conducted on Slovenian milk, while geographical region had a smaller effect, although still relevant (Potočnik et al. 2020). Further, summer milk from grass‐fed cattle in Europe is typically characterized by lower saturated FA levels compared to winter milk due to increased consumption of forage crops containing higher contents of polyunsaturated FA (e.g., linoleic acid, 18:2n‐6, and α‐linolenic acid, 18:3n‐3) in summer (Baars et al. 2019; Collomb et al. 2008). Rainfall patterns, altitude, length of grazing season (Potočnik et al. 2020; Baars et al. 2019; Collomb et al. 2008), along with temperature, farming and production practices, and cattle diet and physiology (Mosley et al. 2007; Chilliard et al. 2007; Kadzere et al. 2002) may also affect milk FA content and composition. Regarding production practices, aging of the cheese may also modify its FA profile (Laskaridis et al. 2013) and, in this study, cheese products of different aging levels (e.g., marble and garden herbs cheddar cheese vs old cheddar) were provided to the participants in Toronto, while the participants in Halifax were given only one type of cheese (i.e., marble cheese). This difference may have hence further added to the variation between populations.

In this study, the dairy products were provided over a 2‐year period, throughout different seasons, and from two different geographical areas. However, we did not test the presence of any variations along with year and/or season, considering that we do not possess exact information on year and season of production of these samples. Furthermore, even when using the sampling date as a proxy for these two variables, we possess information for products sampled in 2022 only, as no dairy samples were collected in 2021, and relatively low sample sizes for those collected in winter (*n* ≤ 10 and only for the products provided to the participants in Toronto) and no samples at all for the summer and fall seasons for the Toronto population. For these reasons, we were not able to account for these temporal variations when assessing the differences of 15:0, 16:0, and 17:0 levels in the participants' blood.

Considering these factors, and noting that 15:0 and 17:0 were present at relatively low levels in the dairy products, that these FA can be present in other foods, and the variations in adherence rates to the dairy treatments, it is challenging to determine whether these treatments had any specific effect on the participants' levels of 15:0 and 17:0, purely based on FA analysis. In fact, we did not observe any clear trend in the molar content and composition of the target FA in participants' blood, contrary to our initial hypothesis and to what studies have shown (levels of 15:0 and 17:0 rise along with increased dairy fat intake, e.g., Golley and Hendrie 2014). This highlights the importance of more precise techniques, such as compound‐specific stable isotope analysis, and/or combining multiple techniques, as shown in this study (e.g., C‐DHQ II, and FA and isotopic analyses), as well as in Mitchell et al. (2022).

### 4.3 Strengths, Limitations, and Future Directions

This study provides novel and valuable information regarding the dairy FA biomarkers 15:0 and 17:0 in human plasma, as it is the first investigation to look at their stable C isotope composition. However, this study has several limitations that are worth highlighting. First, as mentioned above, this study is a secondary analysis of a project developed to study the effects of full‐fat dairy on lean body mass and resting metabolic rate (Anderson et al. 2026). Therefore, this investigation was purely exploratory and not designed to answer our specific questions, that is, blood levels of 15:0 and 17:0 would be higher in participants exposed to the dairy treatments and their isotopic signature would reflect that of their dairy food sources. With this in mind, results should be regarded with caution. Second, we could not control participants' dietary behaviors during the trial, thus leading to errors related to variable adherence and dietary habits. Whereas it is complex to fully control one's diet for an extended period, such as 12 weeks, even if on a voluntary basis, different measures could be implemented to increase adherence rates in future investigations.

Third, our study included populations of participants from two different geographical locations. This has potentially introduced bias to our analysis, considering that the isotopic signature of the target FA at baseline was significantly different between these two populations. To avoid this issue, future studies should either account a priori for potential geographical and/or major dietary variations at baseline or choose populations that live closer together geographically. Moreover, it would be advisable to control for any other potential spatial and/or temporal variation in the biochemical composition of the dietary treatment and account for any inter‐individual differences in age, sex, ethnicity, social status, among other factors which have been shown to affect the stable C isotope composition in previous studies.

Last, it was not possible to analyze the isotopic composition of other food items containing the target FA in this investigation as we had no control or access to the variety of foods the participants consumed. Nevertheless, this would be strongly advised for future studies to better discriminate between the δ^13^C ratios of 15:0 and 17:0 related to dairy products vs other foods, and to calculate the relative contribution of each food item to the overall C signature (e.g., via mixing models). Furthermore, it would be worthwhile, if possible, to assess the isotopic composition of the target compounds and/or tissues at baseline, prior to starting the dietary treatment, to increase the success of the experiment. While it is known that the stable isotope composition of certain compounds/tissues in consumers reflects that of their food sources (Mitchell et al. 2022; McConnaughey and McRoy 1979; DeNiro and Epstein 1978), and that variations over time can be captured by stable isotope analysis (Klievik et al. 2023; Metherel et al. 2019), this technique is not effective, as we saw for the Toronto population, unless there is a major difference in the isotopic composition at baseline between the food source and its consumer(s).

These limitations are just a few examples of the many challenges (e.g., genetic and life‐style variability, Hedrick et al. 2012, Jenab et al. 2009; equipment high‐cost and accessibility) that may be encountered while trying to identify and validate nutritional biomarkers. Biomarkers may represent excellent tools and indicators of dietary intake and nutrient status when appropriately selected. While more potential nutritional biomarker candidates are identified thanks to the improved technology (Frangogiannis 2012), more thorough work is necessary to assess their validity, reliability, and sensitivity (Hedrick et al. 2012, Jenab et al. 2009). In this study, although we faced several limitations (listed above) while trying to validate the dairy fatty acid biomarkers 15:0 and 17:0 using compound‐specific stable isotope analysis, we do think that this technique is promising and powerful and that more appropriately tailored studies are needed.

## 5 Conclusions

In this investigation, we found that the isotopic signatures of plasma 15:0 and 17:0 were more enriched in the high‐adherence group of participants in Halifax who consumed dairy products (especially the DCR arm), and that these ratios were more similar to the signatures of the dairy food sources, as hypothesized, thus reflecting dairy consumption. In contrast, against one of our initial hypotheses, no specific trends were observed when comparing the molar content and composition of the target FA in participants' blood.

We also observed major differences in the molar content and composition, as well as in the isotopic signature of the target FA between the participants in Halifax vs Toronto, which may be related to the different adherence rates, as well as to intrinsic geographic and dietary variations between the two populations, as well as inter‐individual differences. In this regard, the lack of variation in the isotopic signature of the target FA in the Toronto participants could be due to the fact that their baseline δ^13^C ratios were more enriched than those measured in the participants in Halifax, thus masking any potential effect of the dairy treatment.

Last, we detected a few differences, although relatively minor, especially for 15:0 and 17:0, in the FA content and composition of dairy products given to participants in Halifax vs Toronto. Considering that these products were produced over different seasons and provided to the participants over different months, spatial and temporal variations were expected although we were not able to assess the temporal ones.

Overall, these findings show how compound‐specific stable isotope analysis may provide more accurate information on the dairy FA biomarkers and the potential of this technique to not only assess dairy consumption, but also to better clarify the association between dairy foods and chronic diseases in future studies. Furthermore, it highlights major gaps that should be addressed in future investigations and the importance of combining multiple techniques for a better understanding of the results.

## Author Contributions

Author C.P. was responsible for data curation, formal analysis, investigation, visualization, and writing the original draft. G.H.A., P.K., and B.L. provided support in the design and development of methodology of the clinical trial, whereas J.C. and A.J.H. assisted with the analysis of the DHQ data. All the coauthors reviewed and edited the draft. Last, R.P.B. provided supervision, as well as resources and funds for this project.

## Funding

This study was funded by the Dairy Farmers Association of Canada (DFC) awarded to RP Bazinet on Sept 17, 2021, with AJ Hanley and GH Anderson as co‐applicants. The DFC association had no role in the design, analysis, or writing of this article.

## Conflicts of Interest

R.P.B. has received industrial grants, including those matched by the Canadian government, and/or travel support or consulting fees largely related to work on brain fatty acid metabolism or nutrition from Arctic Nutrition, Bunge Ltd., Dairy Farmers of Canada, DSM, Fonterra Inc., Mead Johnson, Natures Crops International, Nestec Inc., Pharmavite, Sancero Inc., and Spore Wellness Inc. Moreover, R.P.B. is on the executive of the International Society for the Study of Fatty Acids and Lipids and held a meeting on behalf of Fatty Acids and Cell Signaling, both of which rely on corporate sponsorship. Last, R.P.B. has given expert testimony in relation to supplements and the brain. The authors C.P., M.S., S.V., J.‐E.C., A.J.H., P.K., B.L., and G.H.A. declare none.

## Supporting information


**Appendix S1:** List of exclusion criteria.
**Figure S2:** Flowchart of the six phases of the original parallel, multi‐site, randomized clinical study, from where the samples for this secondary analysis were obtained.
**Figure S3:** Variations in the mean consumption rate (as serving/day), over the past month, of food items known to contain the target dairy fatty acid biomarkers 15:0 and 17:0 (i.e., ‘milk beverages’, ‘yogurt’, ‘cheese’, ‘dairy products’, ‘meat’, ‘fish/seafood’, ‘eggs’) and food items known to affect the consumers' bulk δ^13^C signature (i.e., ‘sugary drinks', ‘corn’, ‘legumes', ‘rice/other grains', ‘breakfast cereals/breads') between the participants in Halifax and Toronto at baseline. Consumption rates were calculated based on the information obtained through the the Past‐Month Canadian Diet History Questionnaire II filled up by the participants prior to the trial, as described within the Materials and Methods section, as well as Appendix 2. Bars represent standard deviation, whereas an asterisk indicates a significant difference (*p* < 0.05) in the consumption of ‘legumes' between the Halifax and Toronto participants following upaired *t*‐test.
**Figure S4:** Variations in the mean consumption rate (as serving/day), over the past month, of milk, yogurt, and cheese products between the participants in Halifax and Toronto at weeks 0 and 12, and across the calorie restricted (CR), dairy (D), and dairy‐calorie restricted (DCR) treatments. Consumption rates were calculated upon the information obtained through the Past‐Month Canadian Diet History Questionnaire II filled up by the participants at the start and end of the trial, as described within the Materials and Methods section, as well as Appendix S4. Bars represent standard deviation.
**Appendix S5:** Calculation of food consumption from the Past‐Month Canadian Diet History Questionnaire II.
**Appendix S6:** List of the dairy products provided to the participants.
**Table S7:** Results of the permutational analysis of variance (PERMANOVA) performed to assess whether there were any effects of sampling site (Halifax, Toronto) and treatment (i.e., calorie restricted; dairy; dairy and calorie restricted) on plasma and red blood cell (RBC) content and composition, as well as plasma isotopic composition of 15:0, 16:0, and 17:0. Plasma and RBC FA content data were log transformed (Log(x + 1)) prior to creating the Bray‐Curtis resemblance matrices, whereas plasma and RBC FA composition data were square root transformed. For the plasma isotopic composition data, we used Euclidean distance to build resemblance matrices due to the negative values. All PERMANOVA tests were run with unrestricted permutation of raw data and type III sums of square (9999 number of permutations), and we used ‘Sampling site’ (random) and ‘Treatment’ (fixed) as the ‘Factors’ in the PERMANOVA design. Asterisks indicate different levels of statistical significance, with the number of asterisks corresponding to specific *p* value thresholds, i.e., *, ≤ 0.05; **, ≤ 0.01; ***, ≤ 0.001.
**Table S8:** Number (*n*) of servings of milk, yogurt, and cheese servings provided to each participant in Halifax, along with the number of servings consumed and their relative proportions (%). Start and end date of the trial period are also shown. Overall mean proportions are highlighted in orange. Red characters differentiate participants who showed less than 89% adherence.
**Table S9:** Number (*n*) of servings of milk, yogurt, and cheese servings provided to each participant in Toronto, along with the number of servings consumed and their relative proportions (%). Start and end date of the trial period are also shown. Overall mean proportions are highlighted in orange. Red characters differentiate participants who showed less than 79% adherence.
**Table S10:** Mean molar amounts (μmol/L) and percentages (mol%) ± SD of 15:0, 16:0, and 17:0 measured in the plasma of the participants in Halifax and Toronto collected at weeks 0, 4, 8, and 12 and across the various dietary treatments (i.e., calorie restricted, CR; dairy, D; dairy and calorie restricted, DCR). Sample sizes (n) are also shown.
**Table S11:** Mean molar amounts (nmol/g) and percentages (mol%) ± SD of 15:0, 16:0, and 17:0 measured in the red blood cells of the participants in Halifax and Toronto collected at weeks 0, 4, 8, and 12 and across the various dietary treatments (i.e., calorie‐restricted, CR; dairy, D; dairy and calorie restricted, DCR). Sample sizes (n) are also shown.
**Figure S12:** Mean δ^13^C ratios of 15:0 (orange circles), 16:0 (green squares), and 17:0 (blue triangles) measured in random subsamples of the dairy products (i.e., cheese, milk, and yogurt combined) provided to the participants in Halifax and Toronto. Error bars represent standard errors (n_H_ = 9, n_T_ = 9).
**Table S13:** Results of one‐way analysis of variance (ANOVA) tests performed to assess the differences across treatments in the δ^13^C ratios of plasma 15:0, 16:0, and 17:0 measured in the participants in Halifax and Toronto.
**Table S14:** Mean δ^13^C ratios (mUR) and standard deviation of 15:0, 16:0, and 17:0 measured in the plasma of the participants in Halifax and Toronto collected at weeks 0, 4, 8, and 12 and across the various dietary treatments (i.e., calorie‐restricted, CR; dairy, D; dairy and calorie restricted, DCR). Sample sizes (n) are also reported.
**Figure S15:** Mean δ^13^C ratios of 15:0 (orange circles), 16:0 (green squares), and 17:0 (blue triangles) measured in the plasma of the participants in low‐ vs. high‐adherence groups of participants in Halifax and Toronto, across the calorie restricted (CR), dairy (D), and dairy‐calorie restricted (DCR) treatments. Low‐ and high‐adherence groups were established according to the relative median proportion of adherence of the participants to the assigned dairy treatments. Samples at weeks 4, 8, and 12 were grouped for each treatment. Error bars represent standard errors (n_H_Low_ = 15–28, n_H_High_ = 11–28, n_T_Low_ = 20–44, n_T_High_ = 12–44), while a letter code highlights significant (*p* < 0.05) Tukey's pairwise comparisons across treatments following one‐way analysis of variance (Table S12), and dashed lines identifies the δ^13^C ratios of 15:0 (orange), 16:0 (green), and 17:0 (blue) measured in the dairy items provided to the participants.
**Table S16:** Results of one‐way analysis of variance (ANOVA) tests performed to assess the differences across treatments in the δ^13^C ratios of plasma 15:0, 16:0, and 17:0 measured in low‐ vs. high‐adherence groups of participants in Halifax and Toronto. Low‐ and high‐adherence groups were established according to the relative median proportion of adherence of the participants to the assigned dairy treatments (i.e., 89% for the Halifax participants, 79% for the Toronto participants).

## Data Availability

The data that support the findings of this study are available from the corresponding author upon reasonable request.
