# Supplementary material for: Compound‐Specific Stable Isotope Analysis Improves the Association Between Dairy Fatty Acid Biomarkers and Dairy Intake: A Secondary Analysis
Source: Lipids. 2026 Apr 1;61(4):537–50. doi: 10.1002/lipd.70053 (PMC13341346; doi:10.1002/lipd.70053)
Supplement: Supplementary file 1 — Appendix S1: List of exclusion criteria. Figure S2: Flowchart of the six phases of the original parallel, multi‐site, randomized clinical study, from where the samples for this secondary analysis were obtained. Figure S3: Variations in the mean consumption rate (as serving/day), over the past month, of food items known to contain the target dairy fatty acid biomarkers 15:0 and 17:0 (i.e., ‘milk beverages’, ‘yogurt’, ‘cheese’, ‘dairy products’, ‘meat’, ‘fish/seafood’, ‘eggs’) and food items known to affect the consumers' bulk δ13C signature (i.e., ‘sugary drinks', ‘corn’, ‘legumes', ‘rice/other grains', ‘breakfast cereals/breads') between the participants in Halifax and Toronto at baseline. Consumption rates were calculated based on the information obtained through the the Past‐Month Canadian Diet History Questionnaire II filled up by the participants prior to the trial, as described within the Materials and Methods section, as well as Appendix 2. Bars represent standard deviation, whereas an asterisk indicates a significant difference (p < 0.05) in the consumption of ‘legumes' between the Halifax and Toronto participants following upaired t‐test. Figure S4: Variations in the mean consumption rate (as serving/day), over the past month, of milk, yogurt, and cheese products between the participants in Halifax and Toronto at weeks 0 and 12, and across the calorie restricted (CR), dairy (D), and dairy‐calorie restricted (DCR) treatments. Consumption rates were calculated upon the information obtained through the Past‐Month Canadian Diet History Questionnaire II filled up by the participants at the start and end of the trial, as described within the Materials and Methods section, as well as Appendix S4. Bars represent standard deviation. Appendix S5: Calculation of food consumption from the Past‐Month Canadian Diet History Questionnaire II. Appendix S6: List of the dairy products provided to the participants. Table S7: Results of the permutational analysis of variance (PE [file LIPD-61-537-s001.docx]

**Supplementary Information**

**Compound-specific stable isotope analysis improves the association between dairy fatty acid biomarkers and dairy intake: a secondary analysis**

Camilla Parzanini^1^, Marisa Soo^1^, Shirley Vien^1^, Ji-Eun Chon^1^, Anthony J. Hanley^1^, Priya Kathirvel^2^, Bohdan Luhovyy^2^, G. Harvey Anderson^1^, Richard P. Bazinet^1^

^1^Department of Nutritional Sciences, University of Toronto, Toronto, ON, Canada

^2^Mount Saint Vincent University, Halifax, NS, Canada

**Appendix S1 List of exclusion criteria**

- Fasting blood glucose ≥ 7 mmol/L.

- Triglycerides ≥ 2.3 mmol/L (measured not until the first study visit).

- Usage of smoking tobacco products, marijuana, and edibles > 1 to 2 time/month.

- Thyroid issues.

- Previous history of cardiovascular disease, diabetes, liver or kidney disease, inflammatory bowel disease, celiac disease, short bowel syndrome, any malabsorptive syndrome, pancreatitis, gallbladder, or biliary disease.

- Presence of gastrointestinal disorder or surgeries within the past year.

- Consuming prescription or non-prescription drug, herbal or nutritional supplements known to affect blood glucose or that could affect the outcome of the study as per investigator’s judgement.

- Known to be pregnant or lactating or planning on becoming pregnant in the next 12 months.

- Irregular menstrual cycles (i.e. frequent missed cycles), undergoing menopausal symptoms.

- Unwillingness or inability to comply with the experimental procedures.

- Known intolerance, sensitivity, and/or allergy to dairy products.

- Consumption of protein powders/protein supplements.

- Extreme dietary habits (i.e., Atkins diet, very high protein diets, etc.).

- Uncontrolled hypertension (systolic blood pressure ≥ 140 mm Hg or diastolic blood pressure ≥ 90 mm Hg) as defined by the average blood pressure measured at screening.

- Weight gain or loss of at least 10 lbs (~4.5 kg) in previous three months, and history of childhood overweight or obesity.

- Excessive alcohol intake (i.e., more than 2 drinks per day or more than 9 drinks per week).


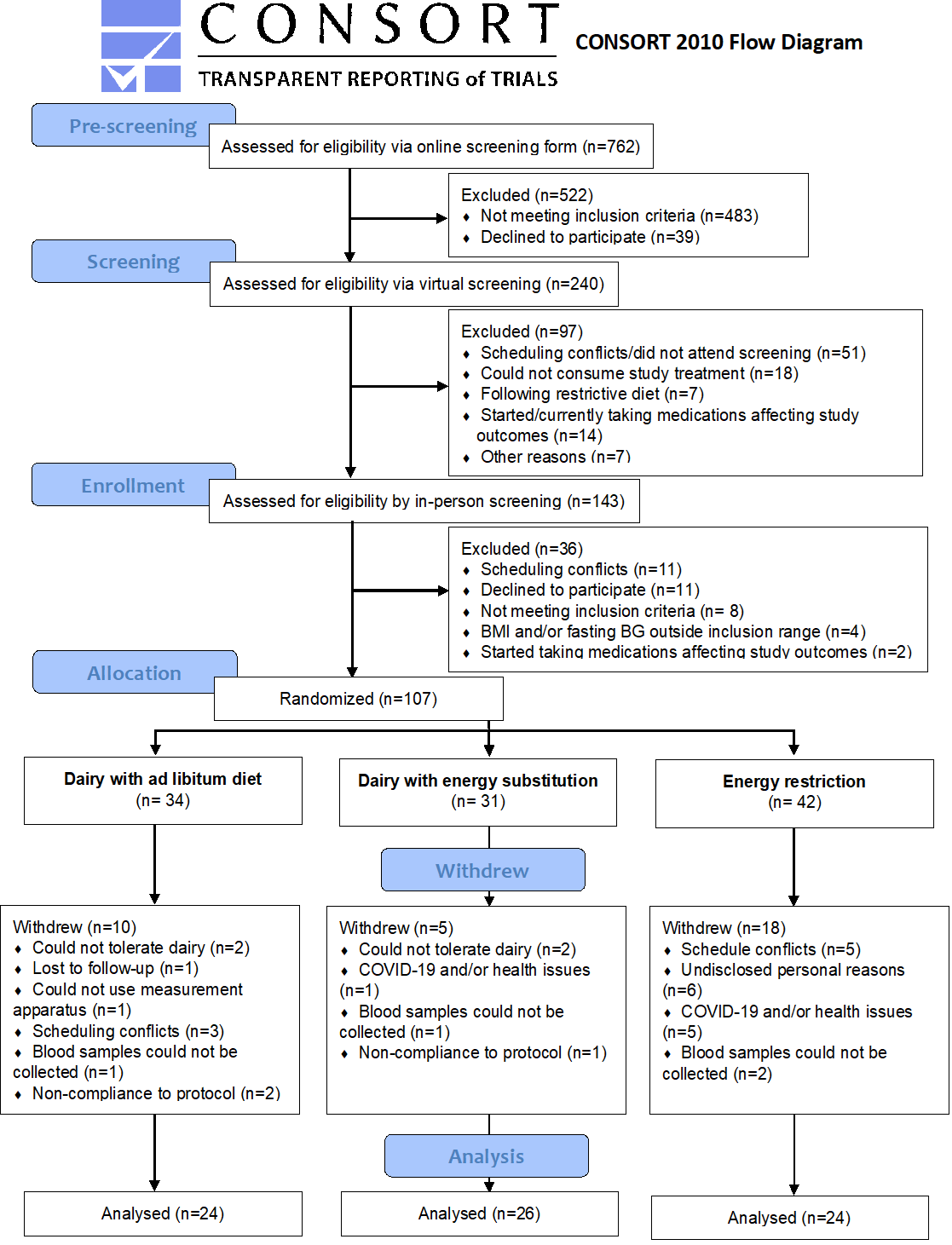


**Figure S2** Flowchart of the six phases of the original parallel, multi-site, randomized clinical study, from where the samples for this secondary analysis were obtained.

**
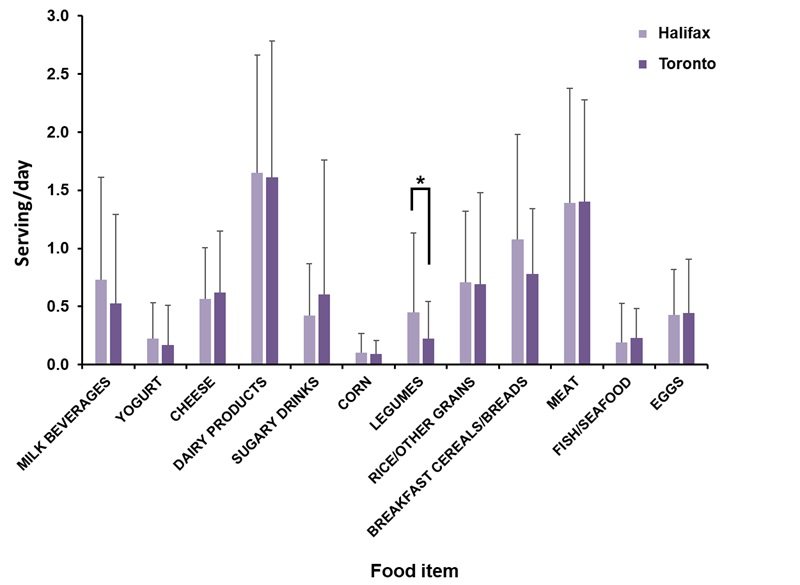
**

**Figure S3** Variations in the mean consumption rate (as serving/day), over the past month, of food items known to contain the target dairy fatty acid biomarkers 15:0 and 17:0 (i.e., ‘milk beverages’, ‘yogurt’, ‘cheese’, ‘dairy products’, ‘meat’, ‘fish/seafood’, ‘eggs’) and food items known to affect the consumers’ bulk δ^13^C signature (i.e., ‘sugary drinks’, ‘corn’, ‘legumes’, ‘rice/other grains’, ‘breakfast cereals/breads’) between the participants in Halifax and Toronto at baseline. Consumption rates were calculated based on the information obtained through the the Past-Month Canadian Diet History Questionnaire II filled up by the participants prior to the trial, as described within the Materials and Methods section. Bars represent standard deviation, whereas an asterisk indicates a significant difference (p < 0.05) in the consumption of ‘legumes’ between the Halifax and Toronto participants following upaired t-test.

**
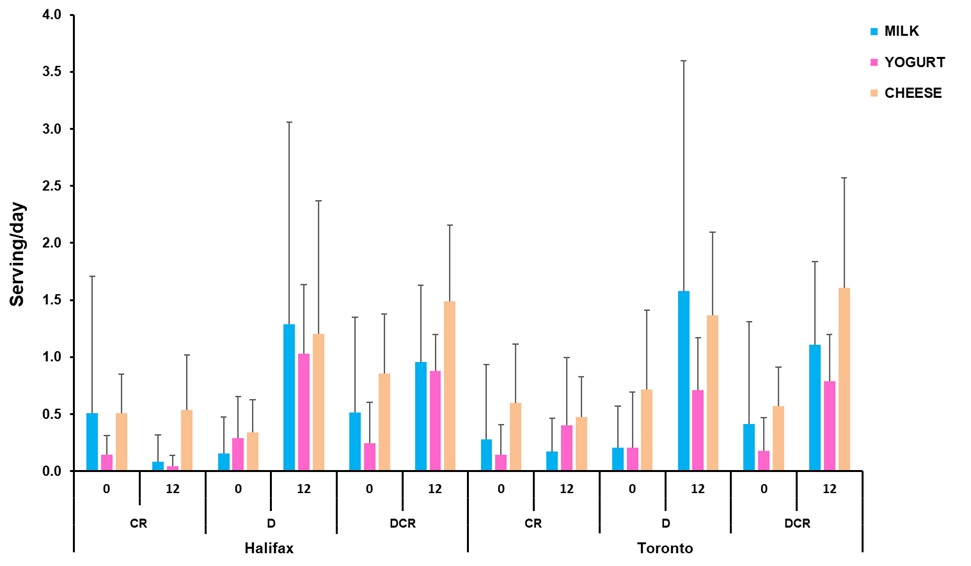
**

**Figure S4** Variations in the mean consumption rate (as serving/day), over the past month, of milk, yogurt, and cheese products between the participants in Halifax and Toronto at weeks 0 and 12, and across the calorie restricted (CR), dairy (D), and dairy-calorie restricted (DCR) treatments. Consumption rates were calculated upon the information obtained through the Past-Month Canadian Diet History Questionnaire II filled up by the participants at the start and end of the trial, as described within the Materials and Methods section. Bars represent standard deviation.

**Appendix S5 Calculation of food consumption from the Past-Month Canadian Diet History Questionnaire II**

The Diet History Questionnaire provided to the participants was a modified version of the Past-Month Canadian Diet History Questionnaire II (C-DHQ II) from the U.S. National Cancer Institute (https://epi.grants.cancer.gov/dhq2/). The C-DHQ II was provided prior to starting the trial, as well as at the end at week 12. Modifications were made to include questions about dairy milk alternatives (e.g. almond and coconut milk). While these questions are present in the version III of the DHQ for U.S., the C-DHQ III counterpart has not been released yet. Due to these changes to the DHQ, it was not possible to obtain the complete nutrient and food group intake estimates from the participants using the Diet*Calc software available on the website provided above. Nonetheless, we calculated the consumption rate, over the past month, of those food items that are known to include the target FA, in addition to cheese, milk, and yogurt, along with other food items that may affect the bulk δ^13^C signature. Specifically, we used frequency information (e.g., “How often in the past month...”) and quantities of foods (e.g., cups, ounces), converted into “servings” based upon the questionnaire answers, as well as the nutrition labels of Canadian or North American products, to measure consumption as serving/day. Ranges of frequency options and quantities varied across food items. A PDF file with the original file of the C-DHQ- II is available (<https://epi.grants.cancer.gov/dhq2/forms/dhq2_pastmonth_noportion.pdf>), and may be used to obtain a picture of these ranges. Once the answers were converted into numerical values (serving/day), groups of similar food items were pooled together to create specific food categories which are the ones plotted in Supplementary Figures S1 and S2, such as ‘milk beverages’, ‘yogurt’, ‘cheese’, ‘dairy products’, ‘sugary drinks’, ‘corn’, ‘legumes’, ‘rice/other grains’, ‘breakfast cereals/breads’, ‘meat’, ‘fish/seafood’ and ‘eggs’. Specifically, the resulting pooled consumption rate was calculated as the sum of all the individual rates of the items included in each given category, except for ‘dairy products’ which was calculated as the sum of ‘milk beverages’, ‘yogurt’, ‘cheese’, and ‘other dairy’. A series of unpaired t-tests were then run to assess the difference in the consumption rate of each food category between the participants in Halifax vs Toronto. All the questions, conversions, and individual food items used to build the final results are provided in the table below.

| **Food category** | **C-DHQ-II question** | **Conversion formula** |
| --- | --- | --- |
| **DAIRY MILK BEVERAGES** | Over the past month, how often did you drink milk as a beverage (NOT in coffee, tea, or cereal)? Do NOT include chocolate milk, hot chocolate, and milk in milkshakes or meal re... | 1 cup = 1 serving |
|  | Each time you drank milk as a beverage, how much did you usually drink? |  |
|  | Over the past month, how often did you drink chocolate milk or hot chocolate? | 1 cup = 1 serving |
|  | Each time you drank chocolate milk or hot chocolate, how much did you usually drink? |  |
|  | Over the past month, how often did you drink milkshakes? | 1 cup (8 oz) = 1 serving |
|  | Each time you drank milkshakes, how much did you usually drink? |  |
|  | Over the past month, how often did you eat oatmeal, Cream of Wheat, Red River, or other cooked cereal? | 1 cup = 1 serving |
|  | Each time milk was added to your oatmeal, Cream of Wheat, Red River, or other cooked cereal, how much did you usually add? |  |
|  | Over the past month, how often did you eat cold cereal (including gluten-free)? | 1 cup = 1 serving |
|  | Each time milk was added to your cold cereal, how much did you usually add? |  |
| **SUGARY DRINKS** | Over the past month, how often did you drink soft drinks or pop? | 1 regular size can or bottle (355 mL; 12-16oz) = 1 serving |
|  | Each time you drank soft drinks or pop, how much did you usually drink? |  |
|  | Over the past month, how often did you drink beer? | 1 bottle (473 ml) = 1 serving |
|  | Each time you drank beer, how much did you usually drink? |  |
|  | Over the past month, how often did you drink wine or wine coolers? | 1 glass (5 oz) = 1 serving |
|  | Each time you drank wine or wine coolers, how much did you usually drink? |  |
|  | Over the past month, how often did you drink liquor or mixed drinks? | 1 shot = 1 serving |
|  | Each time you drank liquor or mixed drinks, how much did you usually drink? |  |
| **LEGUMES** | Over the past month, how often did you eat string beans or green beans (including fresh, canned, or frozen)? | 1 cup = 1 serving |
|  | Each time you ate string beans or green beans, how much did you usually eat? |  |
|  | Over the past month, how often did you eat peas (including fresh, canned, or frozen)? | 1/2 cup = 1 serving |
|  | Each time you ate peas, how much did you usually eat? |  |
|  | Over the past month, how often did you eat cooked dried or canned beans (such as baked beans, pintos, lentils, kidney, black-eyed peas, lima, soybeans, refried beans, or others)? Do NOT include be... | 1/2 cup = 1 serving |
|  | Each time you ate cooked dried or canned beans, how much did you usually eat? |  |
|  | Over the past month, how often did you eat baked beans (including canned, ready-made, or homemade)? | 1/2 cup = 1 serving |
|  | Each time, you ate baked beans, how much did you usually eat? |  |
| **CORN** | Over the past month, how often did you eat corn (including fresh, canned, or frozen)? | 1/2 cup = 1 serving |
|  | Each time you ate corn, how much did you usually eat? |  |
| **RICE/OTHER GRAINS** | Over the past month, how often did you eat rice or other cooked grains (such as bulgur, cracked wheat, millet, etc.)? Do NOT include sushi. | 1/2 = 1 serving |
|  | Each time you ate rice or other cooked grains, how much did you usually eat? |  |
| **BREAKFAST CEREALS/BREADS** | Over the past month, how often did you eat oatmeal, Cream of Wheat, Red River, or other cooked cereal? | 1 cup = 1 serving |
|  | Each time you ate oatmeal, Cream of Wheat, Red River, or other cooked cereal, how much did you usually eat? |  |
|  | Over the past month, how often did you eat cold cereal (including gluten-free)? | 1 cup = 1 serving |
|  | Each time you ate cold cereal, how much did you usually eat? |  |
|  | Over the past month, how often did you eat pancakes, waffles, or French toast (including gluten-free)? | 1 to 3 medium pieces = 1 serving |
|  | Each time you ate pancakes, waffles, or French toast, how much did you usually eat? |  |
|  | Over the past month, how often did you eat bagels or English muffins (including gluten-free)? | 1 bagel or English muffin = 1 serving |
|  | Each time you ate bagels or English muffins, how much did you usually eat? |  |
|  | Over the past month, how often did you eat breads, rolls, or flatbreads AS PART OF SANDWICHES OR WRAPS? | 2 slices (1 roll, 1 flatbread) = 1 serving |
|  | Each time you ate breads, rolls, or flatbreads AS PART OF SANDWICHES OR WRAPS, how many did you usually eat? |  |
|  | Over the past month, how often did you eat cornbread or corn muffins? | 1 piece (1 muffin) = 1 serving |
|  | Each time you ate cornbread or corn muffins, how much did you usually eat? |  |
| **MEAT** | Over the past month, how often did you eat roast beef or steak IN SANDWICHES OR WRAPS? | 4 ounces = 1 serving |
|  | Each time you ate roast beef or steak IN SANDWICHES OR WRAPS, how much meat did you usually eat? |  |
|  | Over the past month, how often did you eat luncheon or deli-style ham? Do NOT include other types of ham. | 1-3 slices = 1 serving |
|  | Each time you ate luncheon or deli-style ham, how much did you usually eat? |  |
|  | Over the past month, how often did you eat chicken or turkey COLD CUTS (such as loaf, luncheon meat, turkey ham, turkey salami, turkey pastrami, etc.)? Do NOT include other types of turkey or chicken. | 2 slices = 1 serving |
|  | Each time you ate chicken or turkey cold cuts, how much did you usually eat? |  |
|  | Over the past month, how often did you eat other cold cuts or luncheon meats (such as bologna, salami, corned beef, pastrami, etc.)? Do NOT include ham, chicken, or turkey. | 1-3 slices = 1 serving |
|  | Each time you ate other cold cuts or luncheon meats, how much did you usually eat? |  |
|  | Over the past month, how often did you eat hot dogs, wieners, or frankfurters? Do NOT include sausages or vegetarian hot dogs. | 1 hot dog/wiener/frankfurter = 1 serving |
|  | Each time you ate hot dogs, wieners, or frankfurters, how much did you usually eat? |  |
|  | Over the past month, how often did you eat GROUND chicken or turkey? | 4 ounces = 1 serving |
|  | Each time you ate ground chicken or turkey, how much did you usually eat? |  |
|  | Over the past month, how often did you eat baked, broiled, roasted, stewed, grilled, pan-fried, or fried chicken (including chicken nuggets)? Do NOT include chicken in mixed dishes. | 2 drumsticks/wings (1 breast/thigh, 4 to 8 nuggets) |
|  | Each time you ate baked, broiled, roasted, stewed, grilled, pan-fried, or fried chicken, how much did you usually eat? |  |
|  | Over the past month, how often did you eat chicken in mixed dishes (such as salads, sandwiches, casseroles, chicken curries, stews, etc.)? | 1 cup = 1 serving |
|  | Each time you ate chicken in mixed dishes, how much did you usually eat? |  |
|  | Over the past month, how often did you eat turkey (including roast turkey, cutlets, nuggets, sandwiches, in mixed dishes, etc.)? Do NOT include ground turkey. | 2 to 5 ounces = 1 serving |
|  | Each time you ate turkey, how much did you usually eat? |  |
|  | Over the past month, how often did you eat beef hamburgers or cheeseburgers from a FAST FOOD or OTHER RESTAURANT? | 1 burger = 1 serving |
|  | Each time you ate beef hamburgers or cheeseburgers from a fast food or other restaurant, how much did you usually eat? |  |
|  | Over the past month, how often did you eat beef hamburgers or cheeseburgers that were NOT from a fast food or other restaurant? | 1 patty = 1 serving |
|  | Each time you ate beef hamburgers or cheeseburgers that were NOT from a fast food or other restaurant, how much did you usually eat? |  |
|  | Over the past month, how often did you eat ground beef in mixtures (such as meatballs, casseroles, chili, meatloaf, etc.)? | 1 cup = 1 serving |
|  | Each time you ate ground beef in mixtures, how much did you usually eat? |  |
|  | Over the past month, how often did you eat beef mixtures (such beef stew, beef curry, beef pot pie, beef and noodles, or beef and vegetables)? | 1/2 cup = 1 serving |
|  | Each time you ate beef mixtures, how much did you usually eat? |  |
|  | Over the past month, how often did you eat roast beef or pot roast? Do NOT include roast beef or pot roast in sandwiches. | 2 to 5 ounces = 1 serving |
|  | Each time you ate roast beef or pot roast, how much did you usually eat? |  |
|  | Over the past month, how often did you eat beef steak? Do NOT include steak in sandwiches. | 3 ounces = 1 serving |
|  | Each time you ate beef steak, how much did you usually eat? |  |
|  | Over the past month, how often did you eat pork or beef spareribs? | 4 ribs = 1 serving |
|  | Each time you ate pork or beef spareribs, how much did you usually eat? |  |
|  | Over the past month, how often did you eat baked ham or ham steak? | 3 ounces = 1 serving |
|  | Each time you ate baked ham or ham steak, how much did you usually eat? |  |
|  | Over the past month, how often did you eat pork (including chops, roasts, in mixed dishes, etc.)? Do NOT include ham, ham steak, or sausage. | 1 chop (2 to 5 ounces) = 1 serving |
|  | Each time you ate pork, how much did you usually eat? |  |
|  | Over the past month, how often did you eat liver (all kinds) or liverwurst? | 3 ounces = 1 serving |
|  | Each time you ate liver or liverwurst, how much did you usually eat? |  |
|  | Over the past month, how often did you eat bacon (all kinds)? | 2 slices = 1 serving |
|  | Each time you ate bacon, how much did you usually eat? |  |
|  | Over the past month, how often did you eat sausage (all kinds)? | 1 large link = 1 serving size |
|  | Each time you ate sausage, how much did you usually eat? |  |
| **FISH/SEAFOOD** | Over the past month, how often did you eat canned tuna or tuna salad (including in sandwiches, casseroles, etc.)? Do NOT include fresh tuna. | 1/4 cup (2 ounces) = 1 serving |
|  | Each time you ate canned tuna or tuna salad, how much did you usually eat? |  |
|  | Over the past month, how often did you eat fresh tuna, trout, anchovy, mackerel, herring, or sardine? | 3 ounces = 1 serving |
|  | Each time you ate fresh tuna, trout, anchovy, mackerel, herring, or sardine, how much did you usually eat? |  |
|  | Over the past month, how often did you eat salmon? | 3.5 ounces = 1 serving |
|  | Each time you ate salmon, how much did you usually eat? |  |
|  | Over the past month, how often did you eat white or lean fish (such as cod, sole, perch, pike, etc.)? Do NOT include ready-to-eat battered fish, fish sticks, or other fried fish. | 4 ounces = 1 serving |
|  | Each time you ate white or lean fish, how much did you usually eat? |  |
|  | Over the past month, how often did you eat ready-to-eat battered fish, fish sticks, or other fried fish, including fast food sandwiches? Do NOT include shellfish. | 1 fillet (2 to 6 ounces) = 1 serving |
|  | Each time you ate ready-to-eat battered fish, fish sticks, or other fried fish, how much did you usually eat? |  |
|  | Over the past month, how often did you eat fried shellfish (such as crab, lobster, shrimp, scallops, clams, etc.)? | 3 ounces = 1 serving |
|  | Each time you ate fried shellfish, how much did you usually eat? |  |
|  | Over the past month, how often did you eat shellfish (such as crab, lobster, shrimp, scallops, clams, etc.) that was NOT fried? | 3 ounces = 1 serving |
|  | Each time you ate shellfish that was not fried, how much did you usually eat? |  |
| **EGGS** | Over the past month, how often did you eat eggs, egg whites, or egg substitutes (including eggs in salads, quiche, souffles, sandwiches, etc.)? Do NOT include eggs in baked goods and desserts. | 2 eggs = 1 serving |
|  | Each time you ate eggs, egg whites, or egg substitutes, how many did you usually eat? |  |
| **YOGURT** | Over the past month, how often did you eat yogurt? Do NOT include frozen yogurt. | 1 container = 1 serving |
|  | Each time you ate yogurt, how much did you usually eat? |  |
| **CHEESE** | Over the past month, how often did you eat cottage cheese or ricotta cheese (including low-fat)? | 1/4 cup = 1 serving |
|  | Each time you ate cottage cheese or ricotta cheese, how much did you usually eat? |  |
|  | Over the past month, how often did you eat cheese (including low-fat; in cheeseburgers, sandwiches, and subs)? Do NOT include cream cheese. | 1 slice (1/2oz) = 1 serving |
|  | Each time you ate cheese, how much did you usually eat? |  |
| **OTHER DAIRY** | Over the past month, how often did you eat whipped cream? | 1 tablespoon = 1 serving |
|  | Each time you ate whipped cream, how much did you usually eat? |  |
|  | Over the past month, how often did you eat frozen yogurt, sorbet, or ices? | 1.5 scoops (3/4 cups) = 1 serving |
|  | Each time you ate frozen yogurt, sorbet, or ices, how much did you usually eat? |  |
|  | Over the past month, how often did you eat ice cream, ice cream bars, or sherbet (including low-fat or fat-free)? | 1 scoop (1/2 cup) = 1 serving |
|  | Each time you ate ice cream, ice cream bars, or sherbet, how much did you usually eat? |  |

**Appendix S6 List of the dairy products provided to the participants**

**Cheese** Armstrong Cheese Sticks in assorted flavours, 31% milk fat MF.

| **Flavour** | **Packaging** | | **Nutrition Facts** | **Ingredients** |
| --- | --- | --- | --- | --- |
| Garden Herbs Cheddar Cheese | 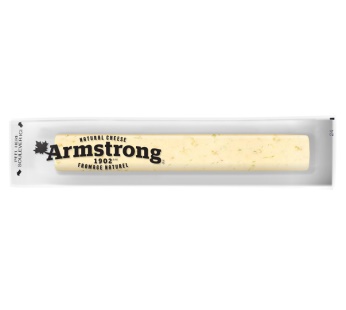 | 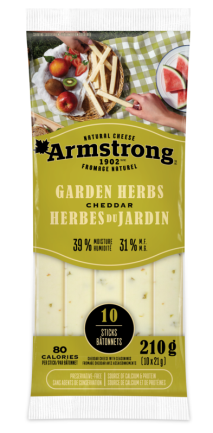 | 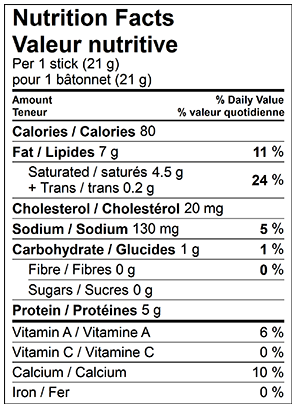 | Pasteurized milk, modified milk ingredients, salt, bacterial culture, calcium chloride, microbial enzyme, seasonings |
| Marble Cheddar | 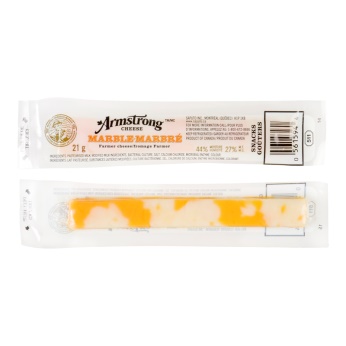 | 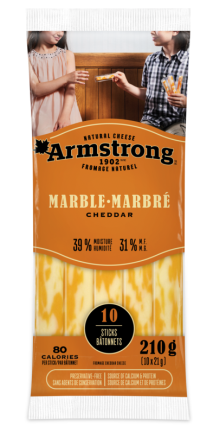 | 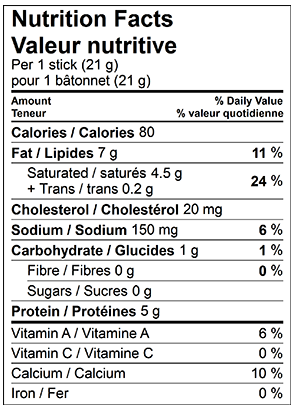 | Pasteurized milk, modified milk ingredients, salt, bacterial culture, calcium chloride, microbial enzyme, colour |
| Old Cheddar | 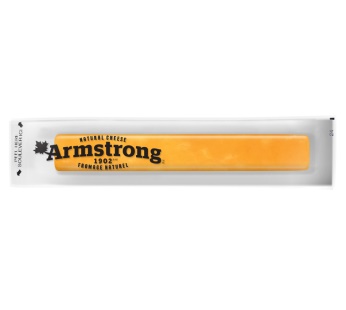 | 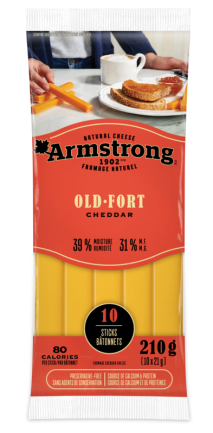 | 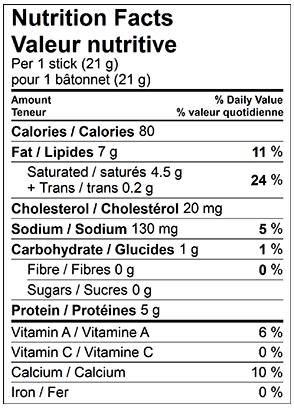 | Pasteurized milk, modified milk ingredients, salt, bacterial culture, calcium chloride, microbial enzyme, colour |

**Milk**

1. Scotsburn Homogenized Milk, 3.25% MF. This product was provided to the participants in Halifax.

| **Packaging** | **Nutrition Facts** | **Ingredients** |
| --- | --- | --- |
| 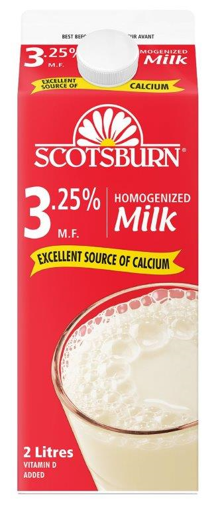 | 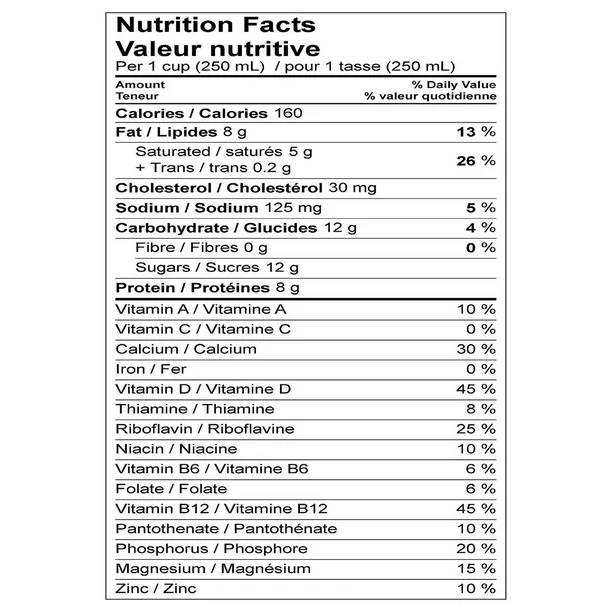 | Pasteurized, homogenized milk, vitamin D_3_ |

1. Neilson, TruTaste Microfiltered Milk, 3.25% MF. This product was provided to the participants in Toronto.

| **Packaging** | **Nutrition Facts** | **Ingredients** |
| --- | --- | --- |
| 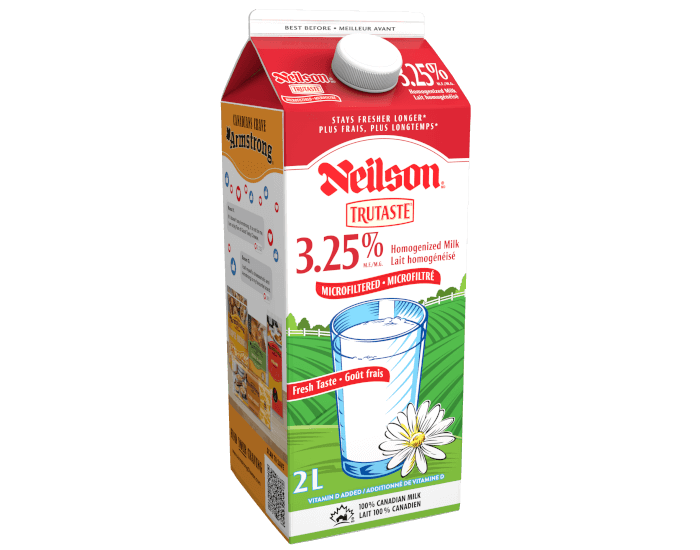 | 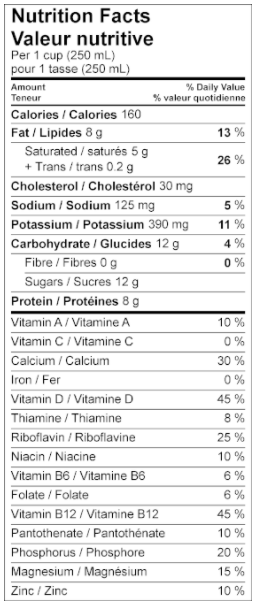 | Microfiltered milk, vitamin D­_3_ |

**Yogurt** Danone Oîkos Yogurt in assorted flavours, 2% MF.

| **Flavour** | **Packaging** | **Nutrition Facts** | **Ingredients** |
| --- | --- | --- | --- |
| Banana | 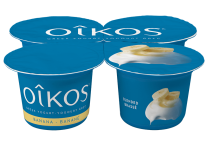 | 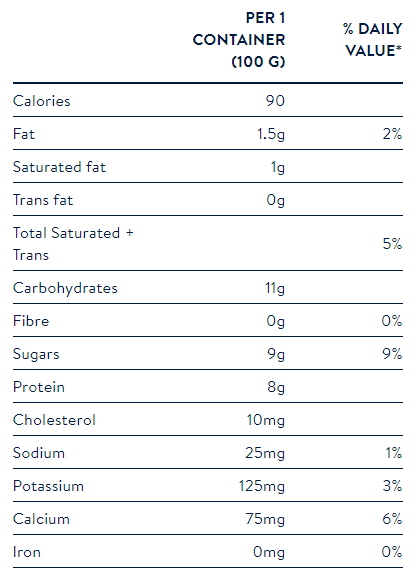 | Yogurt (skim milk, cream, active bacterial cultures)  Banana preparation (water, cane sugar, banana puree, corn starch, natural flavour, lemon juice concentrate, locust bean gum, sodium citrate, carotene [for colour]) |
| Blackberry | 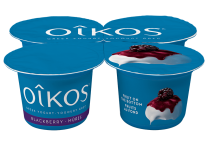 | 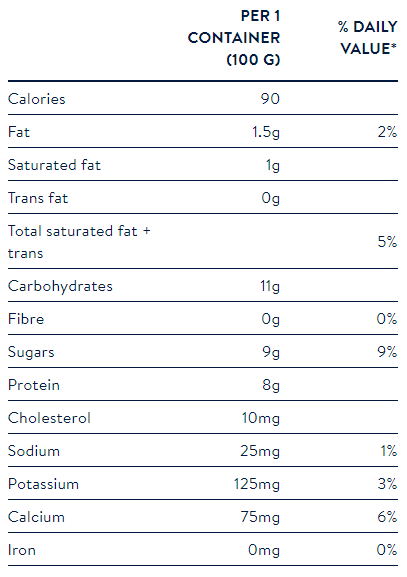 | Yogurt (skim milk, cream, active bacterial cultures)  Fruit on the bottom [sugars (cane sugar, grape juice concentrate and blueberry and carrot juice [for colour]), water, blackberry puree, blackberries, corn starch, natural flavour, citric acid, sodium citrate |
| Blueberry | 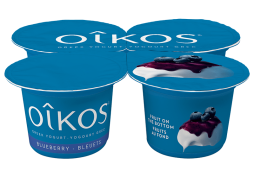 | 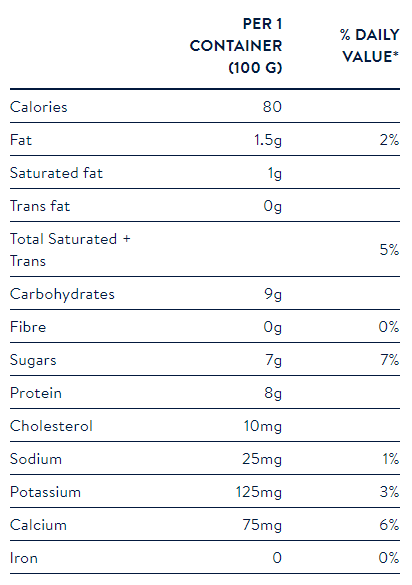 | Yogurt (skim milk, cream, active bacterial cultures)  Fruit on the bottom [water, blueberries, sugars (cane sugar, elderberry juice concentrate [for colour]), corn starch, natural flavour, carrageenan, sodium citrate, lemon juice concentrate |
| Honey | 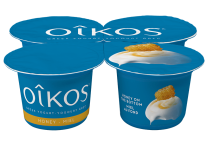 | 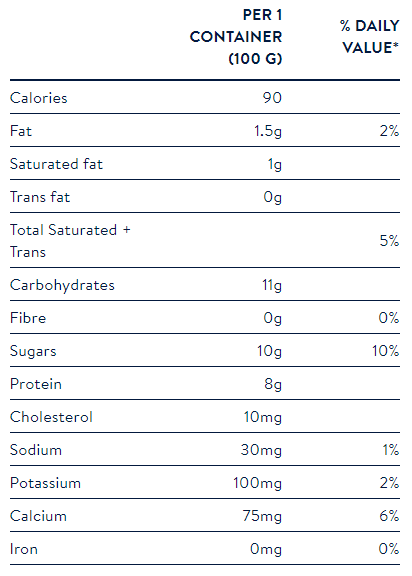 | Yogurt (skim milk, cream, active bacterial cultures)  Honey on bottom [Sugars (honey, cane sugar), water, pectin, natural flavour, corn starch, lemon juice concentrate, calcium citrate, sodium citrate] |
| Key Lime | 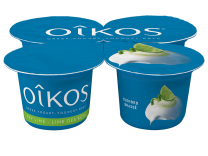 | 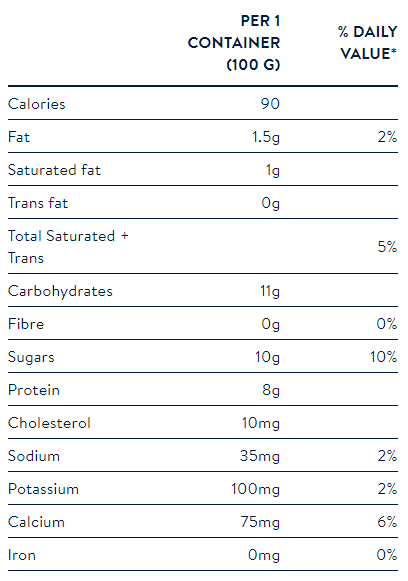 | Yogurt (skim milk, cream, active bacterial cultures)  Key lime preparation [water, sugars (cane sugar, fruit juice concentrate and turmeric [for colour]), corn starch, lime juice concentrate, natural flavour, locust bean gum, sodium citrate] |
| Mandarin Orange | 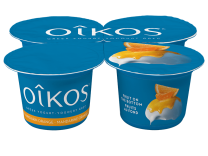 | 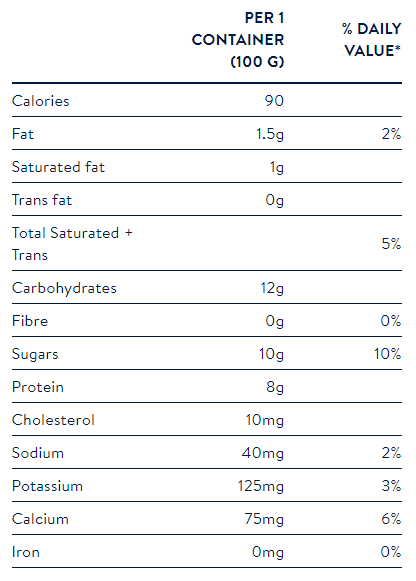 | Yogurt (skim milk, cream, active bacterial cultures)  Fruit on bottom (cane sugar, water, orange puree, orange peel, corn starch, mandarin juice concentrate, lemon juice concentrate, natural flavour, sodium citrate, locust bean gum, black carrot juice, and annatto [for colour]) |
| Passion Fruit | 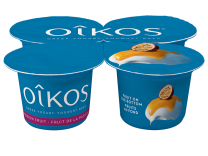 | 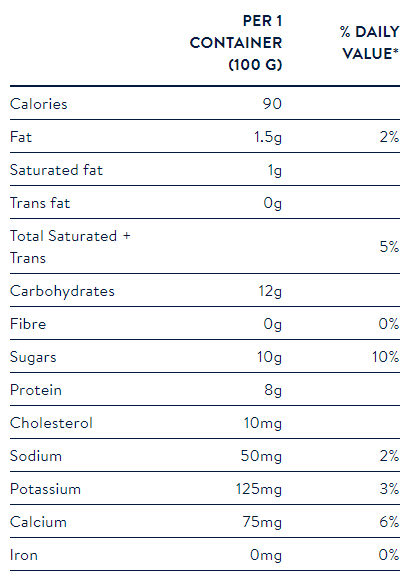 | Yogurt (skim milk, cream, active bacterial cultures)  Fruit on bottom (cane sugar, water, passion fruit juice concentrate, corn starch, sodium citrate, natural flavour, locust bean gum, annatto [for colour], lemon juice concentrate) |
| Pineapple | 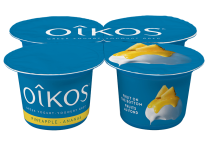 | 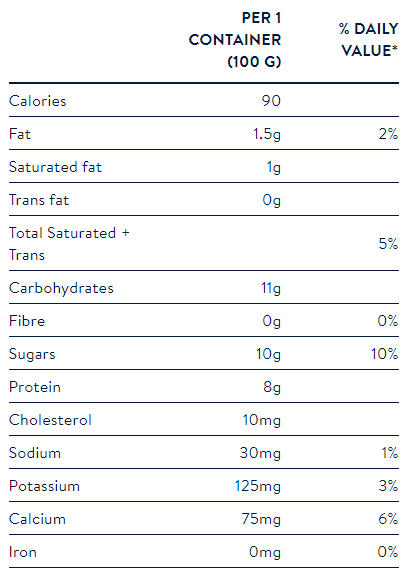 | Yogurt (skim milk, cream, active bacterial cultures)  Fruit on bottom (cane sugar, water, pineapples, pineapple juice concentrate, corn starch, natural flavour, carrageenan, sodium citrate, lemon juice concentrate) |
| Raspberry Pomegranate | 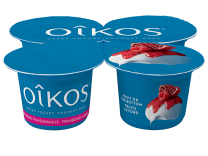 | 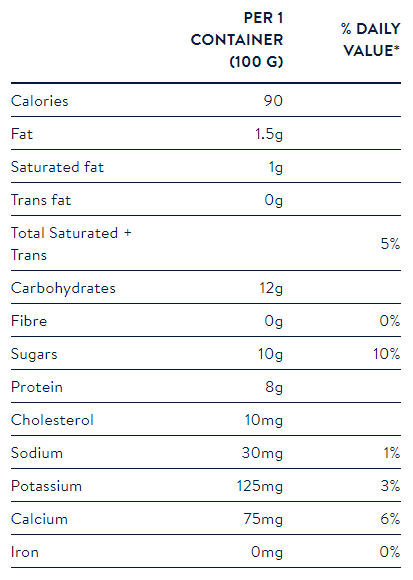 | Yogurt (skim milk, cream, active bacterial cultures)  Fruit on bottom (cane sugar, water, raspberries, corn starch, pomegranate juice concentrate, natural flavour, locust bean gum, black carrot juice [for colour], sodium citrate) |
| Strawberry | 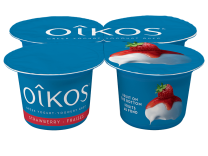 | 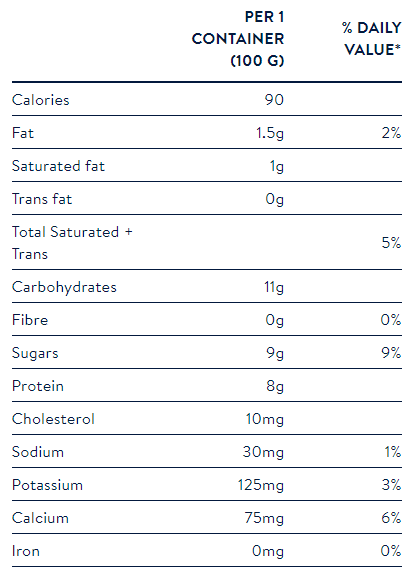 | Yogurt (skim milk, cream, active bacterial cultures)  Fruit on the bottom (sugar, water, strawberries, corn starch, carrageenan, natural flavour, black carrot juice [for colour], sodium citrate, lemon juice concentrate, annatto [for colour]) |
| Strawberry Banana | 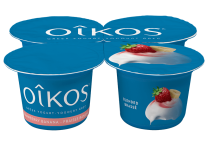 | 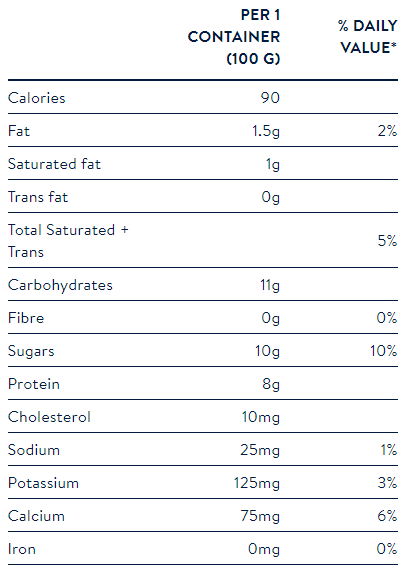 | Yogurt (skim milk, cream, active bacterial cultures)  Strawberry-banana preparation (cane sugar, water, strawberry puree, banana puree, corn starch, locust bean gum, black carrot juice [for colour], natural flavour, lemon juice concentrate) |
| Vanilla | 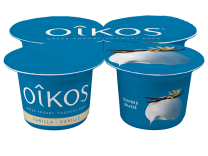 | 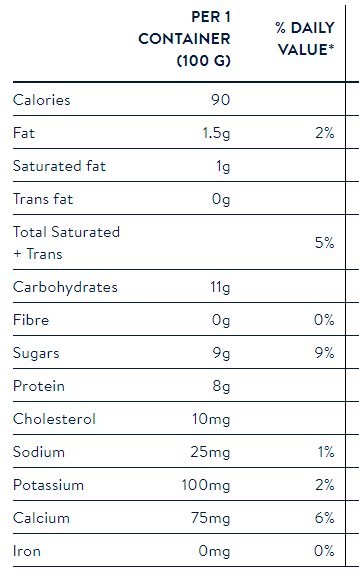 | Yogurt (skim milk, cream, active bacterial cultures)  Vanilla preparation (water, cane sugar, corn starch, natural vanilla flavour, locust bean gum, lemon juice concentrate |

**Table S7** Results of the permutational analysis of variance (PERMANOVA) performed to assess whether there were any effects of sampling site (Halifax, Toronto) and treatment (i.e., calorie restricted; dairy; dairy and calorie restricted) on plasma and red blood cell (RBC) content and composition, as well as plasma isotopic composition of 15:0, 16:0, and 17:0. Plasma and RBC FA content data were log transformed (Log(x+1)) prior to creating the Bray-Curtis resemblance matrices, whereas plasma and RBC FA composition data were square root transformed. For the plasma isotopic composition data, we used Euclidean distance to build resemblance matrices due to the negative values. All PERMANOVA tests were run with unrestricted permutation of raw data and type III sums of square (9999 number of permutations), and we used ‘Sampling site’ (random) and ‘Treatment’ (fixed) as the ‘Factors’ in the PERMANOVA design. Asterisks indicate different levels of statistical significance, with the number of asterisks corresponding to specific p-value thresholds, i.e., *, ≤0.05; **, ≤0.01; ***, ≤0.001.

|  |  | **Sampling Site** | **Treatment** | **Sampling Site x Treatment** |
| --- | --- | --- | --- | --- |
| Plasma FA content |  |  |  |  |
|  | *Pseudo-F* | 7.0874 | 4.7771 | 0.65201 |
|  | *P(perm)* | 0.0032** | 0.0218* | 0.5727 |
|  |  |  |  |  |
| Plasma FA composition |  |  |  |  |
|  | *Pseudo-F* | 38.771 | 0.99411 | 0.80663 |
|  | *P(perm)* | 0.0001*** | 0.6593 | 0.4679 |
|  |  |  |  |  |
| Plasma FA isotopic composition |  |  |  |  |
|  | *Pseudo-F* | 13.208 | 0.69969 | 5.6848 |
|  | *P(perm)* | 0.0001*** | 0.7142 | 0.0002*** |
|  |  |  |  |  |
| RBC FA content |  |  |  |  |
|  | *Pseudo-F* | 20.241 | 2.3496 | 1.8691 |
|  | *P(perm)* | 0.0001*** | 0.2839 | 0.1453 |
|  |  |  |  |  |
| RBC FA composition |  |  |  |  |
|  | *Pseudo-F* | 13.044 | 4.5713 | 0.95865 |
|  | *P(perm)* | 0.0001*** | 0.2016 | 0.4059 |

**Table S8** Number (n) of servings of milk, yogurt, and cheese servings provided to each participant in Halifax, along with the number of servings consumed and their relative proportions (%). Start and end date of the trial period are also shown. Overall mean proportions are highlighted in orange. Red characters differentiate participants who showed less than 89% adherence.

**
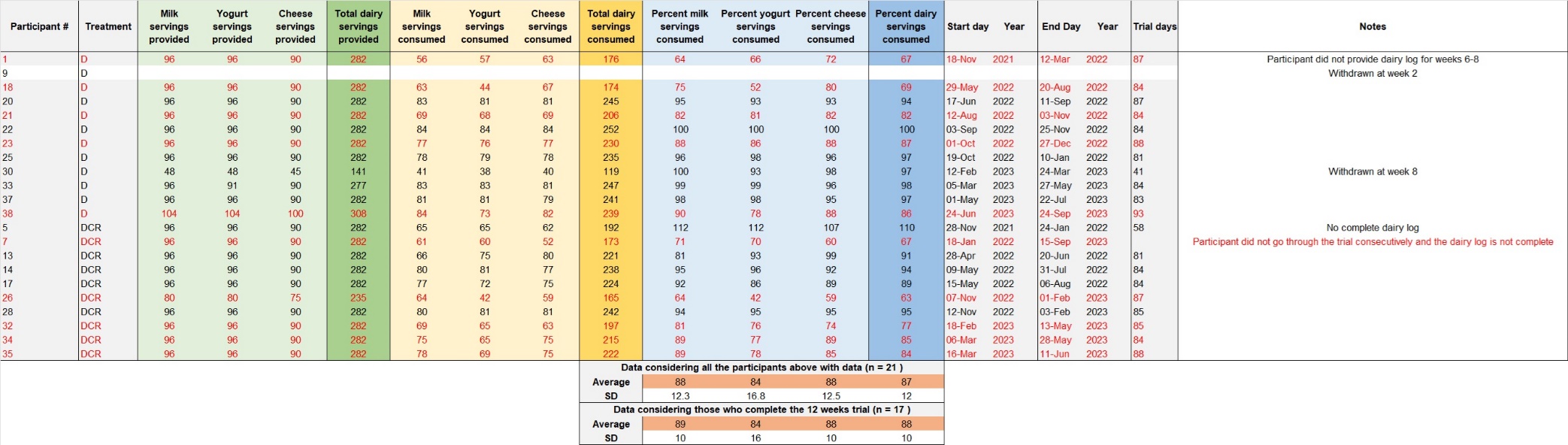
**

**Table S9** Number (n) of servings of milk, yogurt, and cheese servings provided to each participant in Toronto, along with the number of servings consumed and their relative proportions (%). Start and end date of the trial period are also shown. Overall mean proportions are highlighted in orange. Red characters differentiate participants who showed less than 79% adherence.

**
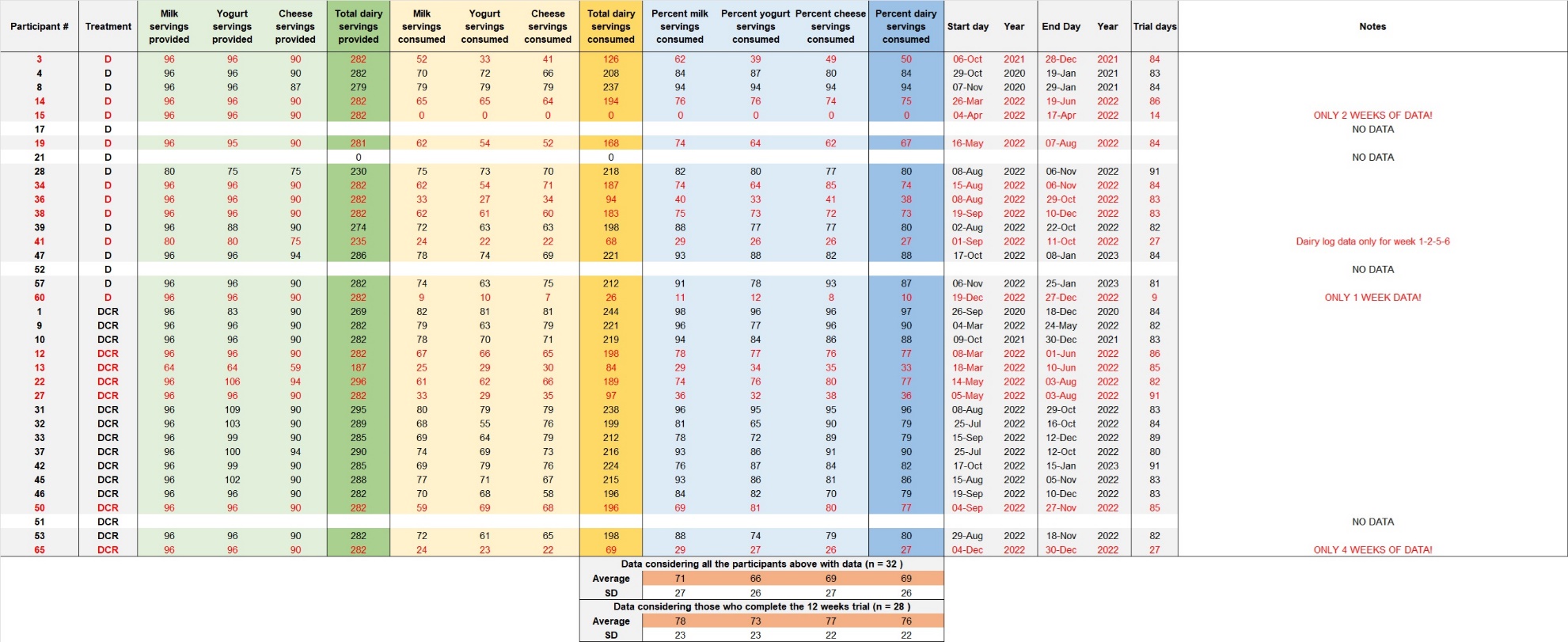
**

**Table S10** Mean molar amounts (μmol/L) and percentages (mol%) ± SD of 15:0, 16:0, and 17:0 measured in the plasma of the participants in Halifax and Toronto collected at weeks 0, 4, 8, and 12 and across the various dietary treatments (i.e., calorie restricted, CR; dairy, D; dairy and calorie restricted, DCR). Sample sizes (n) are also shown.

| **Sampling Site** | **Treatment** | **Week** | **n** | **15:0** | **16:0** | **17:0** | **15:0** | **16:0** | **17:0** |
| --- | --- | --- | --- | --- | --- | --- | --- | --- | --- |
|  |  |  |  | μmol/L | μmol/L | μmol/L | mol% | mol% | mol% |
| **Halifax** |  |  | 126 |  |  |  |  |  |  |
|  | **CR** |  | 40 | 8.19 ± 4.31 | 861.90 ± 205.73 | 13.64 ± 4.65 | 0.24 ± 0.10 | 26.72 ± 2.21 | 0.41 ± 0.08 |
|  |  | 0 | 11 | 7.34 ± 4.95 | 790.61 ± 260.18 | 12.32 ± 5.98 | 0.23 ± 0.10 | 27.28 ± 3.20 | 0.40 ± 0.09 |
|  |  | 4 | 9 | 9.64 ± 5.19 | 960.87 ± 202.32 | 15.42 ± 4.72 | 0.26 ± 0.11 | 27.39 ± 1.61 | 0.43 ± 0.08 |
|  |  | 8 | 10 | 8.60 ± 4.04 | 857.90 ± 194.34 | 14.27 ± 4.20 | 0.26 ± 0.10 | 26.43 ± 1.60 | 0.44 ± 0.08 |
|  |  | 12 | 10 | 7.41 ±2.99 | 855.24 ± 134.02 | 12.87 ± 3.19 | 0.22 ± 0.09 | 25.80 ± 1.75 | 0.39 ± 0.06 |
|  |  |  |  |  |  |  |  |  |  |
|  | **D** |  | 45 | 7.85 ± 2.43 | 1003.51 ± 263.50 | 14.12 ± 3.43 | 0.22 ± 0.06 | 27.61 ± 2.24 | 0.39 ± 0.06 |
|  |  | 0 | 13 | 7.94 ± 2.44 | 995.93 ± 248.64 | 13.57 ± 3.70 | 0.22 ± 0.05 | 27.68 ± 1.96 | 0.38 ± 0.05 |
|  |  | 4 | 11 | 8.29 ± 2.81 | 1023.76 ± 314.07 | 14.02 ± 3.72 | 0.23 ± 0.06 | 27.90 ± 2.27 | 0.39 ± 0.07 |
|  |  | 8 | 11 | 7.91 ± 2.73 | 1006.84 ± 292.59 | 14.15 ± 3.86 | 0.23 ± 0.07 | 28.18 ± 1.95 | 0.40 ± 0.05 |
|  |  | 12 | 10 | 7.20 ± 1.75 | 987.43 ± 226.71 | 14.94 ± 2.47 | 0.20 ± 0.05 | 26.56 ± 2.79 | 0.41 ± 0.07 |
|  |  |  |  |  |  |  |  |  |  |
|  | **DCR** |  | 41 | 9.16 ± 3.97 | 1024.50 ± 356.35 | 14.97 ± 5.61 | 0.24 ± 0.06 | 27.29 ± 2.42 | 0.40 ± 0.08 |
|  |  | 0 | 12 | 8.30 ± 3.71 | 935.05 ± 302.60 | 13.67 ± 5.53 | 0.24 ± 0.05 | 27.35 ± 1.61 | 0.40 ± 0.09 |
|  |  | 4 | 9 | 9.74 ± 3.82 | 986.17 ± 302.86 | 14.86 ± 4.65 | 0.27 ± 0.06 | 27.32 ± 2.20 | 0.41 ± 0.05 |
|  |  | 8 | 10 | 9.31 ± 3.80 | 1029.11 ± 189.04 | 16.07 ± 5.40 | 0.24 ± 0.07 | 26.69 ± 3.87 | 0.41 ± 0.12 |
|  |  | 12 | 10 | 9.53 ± 4.94 | 1161.73 ± 549.64 | 15.53 ± 7.06 | 0.23 ± 0.05 | 27.80 ± 1.69 | 0.38 ± 0.06 |
|  |  |  |  |  |  |  |  |  |  |
| **Toronto** |  |  | 186 |  |  |  |  |  |  |
|  | **CR** |  | 70 | 6.21 ± 2.92 | 898.55 ± 327.04 | 14.51 ± 3.91 | 0.17 ± 0.06 | 24.95 ± 3.92 | 0.42 ± 0.08 |
|  |  | 0 | 22 | 6.29 ± 3.27 | 923.55 ± 403.92 | 14.09 ± 4.64 | 0.17 ± 0.06 | 25.29 ± 4.54 | 0.40 ± 0.09 |
|  |  | 4 | 19 | 6.56 ± 3.60 | 911.29 ± 300.37 | 14.85 ± 3.64 | 0.17 ± 0.08 | 24.90 ± 3.68 | 0.42 ± 0.08 |
|  |  | 8 | 16 | 6.18 ± 2.23 | 919.65 ± 272.31 | 15.40 ± 3.28 | 0.17 ± 0.05 | 24.42 ± 3.56 | 0.42 ± 0.07 |
|  |  | 12 | 13 | 5.57 ± 1.98 | 811.67 ± 302.11 | 13.60 ± 3.81 | 0.18 ± 0.05 | 25.07 ± 3.95 | 0.43 ± 0.05 |
|  |  |  |  |  |  |  |  |  |  |
|  | **D** |  | 52 | 6.60 ± 3.31 | 889.40 ± 382.04 | 15.76 ± 5.83 | 0.18 ± 0.06 | 24.54 ± 4.20 | 0.44 ± 0.08 |
|  |  | 0 | 16 | 6.97 ± 4.36 | 917.42 ± 440.05 | 15.75 ± 7.43 | 0.19 ± 0.08 | 24.40 ± 4.53 | 0.42 ± 0.08 |
|  |  | 4 | 12 | 7.21 ± 3.35 | 985.35 ± 470.99 | 16.95 ± 6.97 | 0.18 ± 0.06 | 24.58 ± 4.91 | 0.43 ± 0.09 |
|  |  | 8 | 13 | 6.19 ± 2.54 | 816.93 ± 312.56 | 15.62 ± 4.06 | 0.18 ± 0.05 | 23.69 ± 4.01 | 0.47 ± 0.08 |
|  |  | 12 | 11 | 5.87 ± 2.41 | 829.60 ± 263.78 | 14.62 ± 3.72 | 0.18 ± 0.05 | 25.69 ± 3.29 | 0.46 ± 0.07 |
|  |  |  |  |  |  |  |  |  |  |
|  | **DCR** |  | 64 | 7.31 ± 4.04 | 1022.71 ± 388.55 | 15.63 ± 4.26 | 0.18 ± 0.05 | 25.05 ± 3.73 | 0.40 ± 0.08 |
|  |  | 0 | 17 | 7.78 ± 3.34 | 1156.91 ± 398.17 | 15.80 ± 4.35 | 0.17 ± 0.04 | 25.19 ± 3.94 | 0.35 ± 0.09 |
|  |  | 4 | 16 | 7.86 ± 3.47 | 1066.43 ± 374.19 | 16.47 ± 4.85 | 0.18 ± 0.04 | 24.84 ± 3.19 | 0.39 ± 0.06 |
|  |  | 8 | 15 | 7.64 ± 6.49 | 934.49 ± 502.93 | 15.44 ± 5.33 | 0.20 ± 0.07 | 24.75 ± 4.02 | 0.43 ± 0.08 |
|  |  | 12 | 16 | 5.96 ± 1.64 | 919.12 ± 217.27 | 14.78 ± 2.08 | 0.17 ± 0.05 | 25.38 ± 4.04 | 0.42 ± 0.08 |

**Table S11** Mean molar amounts (nmol/g) and percentages (mol%) ± SD of 15:0, 16:0, and 17:0 measured in the red blood cells of the participants in Halifax and Toronto collected at weeks 0, 4, 8, and 12 and across the various dietary treatments (i.e., calorie-restricted, CR; dairy, D; dairy and calorie restricted, DCR). Sample sizes (n) are also shown.

| **Sampling Site** | **Treatment** | **Week** | **n** | **15:0** | **16:0** | **17:0** | **15:0** | **16:0** | **17:0** |
| --- | --- | --- | --- | --- | --- | --- | --- | --- | --- |
|  |  |  |  | nmol/g | nmol/g | nmol/g | mol% | mol% | mol% |
| **Halifax** |  |  | 125 |  |  |  |  |  |  |
|  | **CR** |  | 40 | 5.67 ± 1.69 | 1091.64 ± 131.65 | 18.22 ± 2.72 | 0.11 ± 0.03 | 21.77 ± 1.43 | 0.36 ± 0.04 |
|  |  | 0 | 11 | 5.24 ± 1.42 | 1059.51 ± 120.47 | 17.20 ± 2.49 | 0.11 ± 0.03 | 21.38 ± 1.21 | 0.35 ± 0.04 |
|  |  | 4 | 9 | 5.25 ± 1.86 | 1078.62 ± 125.86 | 17.58 ± 2.15 | 0.11 ± 0.04 | 21.99 ± 1.44 | 0.36 ± 0.04 |
|  |  | 8 | 10 | 6.32 ± 1.84 | 1124.82 ± 133.70 | 19.02 ± 2.74 | 0.12 ± 0.03 | 22.03 ± 1.32 | 0.37 ± 0.04 |
|  |  | 12 | 10 | 5.87 ± 1.66 | 1105.51 ± 155.41 | 19.12 ± 3.19 | 0.12 ± 0.03 | 21.72 ± 1.83 | 0.38 ± 0.05 |
|  | **D** |  | 45 | 6.40 ± 1.94 | 1149.95 ± 120.38 | 18.13 ± 3.00 | 0.13 ± 0.04 | 22.57 ± 1.42 | 0.35 ± 0.04 |
|  |  | 0 | 13 | 5.33 ± 1.62 | 1135.78 ± 86.15 | 17.42 ± 2.76 | 0.11 ± 0.03 | 22.51 ± 0.90 | 0.34 ± 0.04 |
|  |  | 4 | 11 | 7.10 ± 2.15 | 1187.24 ± 126.17 | 18.78 ± 3.17 | 0.14 ± 0.04 | 22.80 ± 1.74 | 0.36 ± 0.04 |
|  |  | 8 | 11 | 6.70 ± 1.78 | 1150.36 ± 132.70 | 17.95 ± 2.85 | 0.13 ± 0.04 | 22.72 ± 1.17 | 0.35 ± 0.04 |
|  |  | 12 | 10 | 6.71 ± 1.95 | 1126.91 ± 145.32 | 18.55 ± 3.49 | 0.13 ± 0.04 | 22.24 ± 1.93 | 0.37 ± 0.05 |
|  | **DCR** |  | 40 | 6.37 ± 1.58 | 1083.82 ± 117.94 | 17.71 ± 2.53 | 0.13 ± 0.03 | 21.79 ± 1.29 | 0.36 ± 0.04 |
|  |  | 0 | 12 | 5.99 ± 1.88 | 1095.29 ± 178.29 | 17.76 ± 3.48 | 0.12 ± 0.03 | 21.58 ± 1.67 | 0.35 ± 0.05 |
|  |  | 4 | 9 | 6.80 ± 1.35 | 1078.88 ± 75.84 | 18.08 ± 1.66 | 0.14 ± 0.03 | 21.97 ± 1.02 | 0.37 ± 0.03 |
|  |  | 8 | 10 | 6.30 ± 1.83 | 1057.30 ± 103.29 | 17.51 ± 2.61 | 0.13 ± 0.03 | 21.45 ± 1.31 | 0.36 ± 0.05 |
|  |  | 12 | 9 | 6.52 ± 1.11 | 1102.96 ± 69.50 | 17.52 ± 1.98 | 0.13 ± 0.02 | 22.29 ± 0.90 | 0.35 ± 0.04 |
|  |  |  |  |  |  |  |  |  |  |
| **Toronto** |  |  | 175 |  |  |  |  |  |  |
|  | **CR** |  | 64 | 4.77 ± 1.58 | 1013.85 ± 176.55 | 13.48 ± 5.93 | 0.10 ± 0.03 | 21.21 ± 1.87 | 0.28 ± 0.11 |
|  |  | 0 | 22 | 4.21 ± 1.41 | 950.63 ± 174.62 | 11.81 ± 4.67 | 0.10 ± 0.03 | 21.52 ± 1.73 | 0.27 ± 0.09 |
|  |  | 4 | 17 | 5.35 ± 1.48 | 1094.21 ± 117.44 | 15.06 ± 7.77 | 0.10 ± 0.03 | 21.47 ± 1.46 | 0.30 ± 0.15 |
|  |  | 8 | 14 | 5.16 ± 1.68 | 1063.13 ± 131.68 | 14.52 ± 5.73 | 0.10 ± 0.03 | 20.80 ± 1.60 | 0.28 ± 0.10 |
|  |  | 12 | 11 | 4.50 ± 1.67 | 953.36 ± 246.04 | 13.08 ± 4.91 | 0.10 ± 0.02 | 20.72 ± 2.85 | 0.28 ± 0.08 |
|  |  |  |  |  |  |  |  |  |  |
|  |  |  |  |  |  |  |  |  |  |
|  | **D** |  | 48 | 5.53 ± 1.86 | 1014.49 ± 143.18 | 15.32 ± 3.95 | 0.11 ± 0.03 | 20.97 ± 2.05 | 0.32 ± 0.08 |
|  |  | 0 | 15 | 4.95 ± 2.06 | 983.01 ± 172.56 | 15.00 ± 3.77 | 0.10 ± 0.04 | 20.66 ± 2.13 | 0.31 ± 0.06 |
|  |  | 4 | 12 | 5.69 ± 1.78 | 1021.14 ± 120.93 | 15.32 ± 3.60 | 0.12 ± 0.03 | 20.83 ± 1.87 | 0.31 ± 0.08 |
|  |  | 8 | 11 | 5.92 ± 1.27 | 1031.49 ± 136.53 | 15.63 ± 4.58 | 0.12 ± 0.02 | 20.81 ± 2.10 | 0.32 ± 0.09 |
|  |  | 12 | 10 | 5.78 ± 2.24 | 1035.05 ± 140.05 | 15.46 ± 4.44 | 0.12 ± 0.04 | 21.78 ± 2.19 | 0.32 ± 0.08 |
|  | **DCR** |  | 63 | 5.04 ± 1.75 | 1030.73 ± 155.57 | 14.68 ± 3.68 | 0.10 ± 0.03 | 21.46 ± 1.55 | 0.31 ± 0.06 |
|  |  | 0 | 17 | 4.22 ± 1.44 | 945.60 ± 173.60 | 13.10 ± 3.61 | 0.09 ± 0.02 | 21.21 ± 1.40 | 0.29 ± 0.06 |
|  |  | 4 | 16 | 5.52 ± 1.61 | 1085.85 ± 104.84 | 15.14 ± 3.97 | 0.11 ± 0.03 | 21.63 ± 1.58 | 0.30 ± 0.07 |
|  |  | 8 | 15 | 5.46 ± 1.93 | 1062.52 ± 166.22 | 15.40 ± 3.93 | 0.11 ± 0.04 | 21.44 ± 1.98 | 0.31 ± 0.07 |
|  |  | 12 | 15 | 5.04 ± 1.87 | 1036.64 ± 141.23 | 15.25 ± 2.92 | 0.11 ± 0.04 | 21.59 ± 1.30 | 0.32 ± 0.05 |


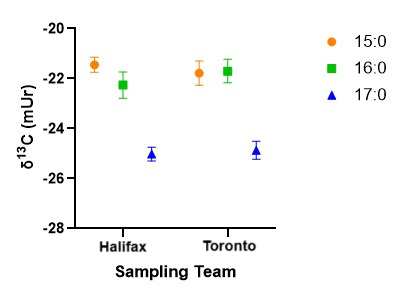


**Sampling Site**

**Figure S12** Mean δ^13^C ratios of 15:0 (orange circles), 16:0 (green squares), and 17:0 (blue triangles) measured in random subsamples of the dairy products (i.e., cheese, milk, and yogurt combined) provided to the participants in Halifax and Toronto. Error bars represent standard errors (n_H_ = 9, n_T_ = 9).

**Table S13** Results of one-way analysis of variance (ANOVA) tests performed to assess the differences across treatments in the δ^13^C ratios of plasma 15:0, 16:0, and 17:0 measured in the participants in Halifax and Toronto.

| **Sampling Site** | **ANOVA table** | **SS** | **DF** | **MS** | **F (DFn, DFd)** | **P value** |
| --- | --- | --- | --- | --- | --- | --- |
| **Halifax** |  |  |  |  |  |  |
|  | **δ^13^C_15:0_** |  |  |  |  |  |
|  | Treatment (between columns) | 31.59 | 2 | 15.80 | F (2, 82) = 3.261 | P=0.0434 |
|  | Residual (within columns) | 397.2 | 82 | 4.4844 |  |  |
|  | Total | 428.8 | 84 |  |  |  |
|  |  |  |  |  |  |  |
|  | **δ^13^C_16:0_** |  |  |  |  |  |
|  | Treatment (between columns) | 31.64 | 2 | 15.82 | F (2, 85) = 13.67 | P<0.0001 |
|  | Residual (within columns) | 98.38 | 85 | 1.157 |  |  |
|  | Total | 130.0 | 87 |  |  |  |
|  |  |  |  |  |  |  |
|  | **δ^13^C_17:0_** |  |  |  |  |  |
|  | Treatment (between columns) | 73.79 | 2 | 36.90 | F (2, 71) = 5.021 | P=0.0091 |
|  | Residual (within columns) | 521.8 | 71 | 7.349 |  |  |
|  | Total | 595.5 | 73 |  |  |  |
|  |  |  |  |  |  |  |
| **Toronto** |  |  |  |  |  |  |
|  | **δ^13^C_15:0_** |  |  |  |  |  |
|  | Treatment (between columns) | 0.7873 | 2 | 0.3937 | F (2, 121) = 0.1480 | P=0.8.626 |
|  | Residual (within columns) | 321.8 | 121 | 2.660 |  |  |
|  | Total | 322.6 | 123 |  |  |  |
|  |  |  |  |  |  |  |
|  | **δ^13^C_16:0_** |  |  |  |  |  |
|  | Treatment (between columns) | 4.413 | 2 | 1.903 | F (2, 121) = 2.185 | P=0.1168 |
|  | Residual (within columns) | 105.4 | 121 | 0.8709 |  |  |
|  | Total | 109.2 | 123 |  |  |  |
|  |  |  |  |  |  |  |
|  | **δ^13^C_17:0_** |  |  |  |  |  |
|  | Treatment (between columns) | 4.173 | 2 | 2.087 | F (2, 121) = 0.7238 | P=0.4870 |
|  | Residual (within columns) | 348.8 | 121 | 2.883 |  |  |
|  | Total | 353.0 | 123 |  |  |  |

**Table S14** Mean δ^13^C ratios (mUR) and standard deviation of 15:0, 16:0, and 17:0 measured in the plasma of the participants in Halifax and Toronto collected at weeks 0, 4, 8, and 12 and across the various dietary treatments (i.e., calorie-restricted, CR; dairy, D; dairy and calorie restricted, DCR). Sample sizes (n) are also reported.

| **Sampling Site** | **Treatment** | **Week** | **n** | **δ^13^C_15:0_** |  | **N** | **δ^13^C_16:0_** |  | **N** | **δ^13^C_17:0_** |
| --- | --- | --- | --- | --- | --- | --- | --- | --- | --- | --- |
|  |  |  |  | mUR |  |  | mUR |  |  | mUR |
| **Halifax** |  |  | 115 |  |  | 121 |  |  | 102 |  |
|  | **CR** |  | 38 | -26.6 ± 2.4 |  | 38 | -25.4 ± 1.2 |  | 33 | -27.9 ± 2.5 |
|  |  | 0 | 10 | -25.8 ± 1.7 |  | 10 | -25.0 ± 1.3 |  | 9 | -27.6 ± 2.2 |
|  |  | 4 | 9 | -26.6 ± 2.1 |  | 9 | -25.8 ± 1.1 |  | 7 | -27.3 ± 1.4 |
|  |  | 8 | 10 | -26.5 ± 2.1 |  | 10 | -25.5 ± 1.3 |  | 9 | -28.8 ± 3.3 |
|  |  | 12 | 9 | -27.8 ± 3.4 |  | 9 | -25.3 ± 1.0 |  | 8 | -27.7 ± 2.8 |
|  |  |  |  |  |  |  |  |  |  |  |
|  | **D** |  | 40 | -26.0 ± 2.4 |  | 44 | -25.1 ± 1.0 |  | 32 | -25.7 ± 2.6 |
|  |  | 0 | 10 | -26.8 ± 3.7 |  | 12 | -25.3 ± 1.1 |  | 8 | -25.0 ± 2.8 |
|  |  | 4 | 10 | -26.0 ± 1.8 |  | 11 | -25.1 ± 0.8 |  | 6 | -26.3 ± 1.4 |
|  |  | 8 | 10 | -25.6 ± 2.1 |  | 11 | -25.2 ± 0.7 |  | 9 | -25.5 ± 1.6 |
|  |  | 12 | 10 | -25.8 ± 1.5 |  | 10 | -24.8 ± 0.8 |  | 9 | -26.0 ± 3.9 |
|  |  |  |  |  |  |  |  |  |  |  |
|  | **DCR** |  | 37 | -25.6 ± 2.2 |  | 39 | -24.2 ± 1.4 |  | 37 | -26.0 ± 2.6 |
|  |  | 0 | 10 | -25.8 ± 2.3 |  | 11 | -24.5 ± 1.6 |  | 11 | -26.4 ± 1.8 |
|  |  | 4 | 8 | -25.5 ± 1.6 |  | 8 | -23.9 ± 1.5 |  | 8 | -26.5 ± 2.1 |
|  |  | 8 | 9 | -24.6 ± 2.2 |  | 10 | -24.1 ± 1.4 |  | 9 | -25.8 ± 3.2 |
|  |  | 12 | 10 | -26.4 ± 2.6 |  | 10 | -24.1 ± 1.3 |  | 9 | -25.3 ± 3.1 |
|  |  |  |  |  |  |  |  |  |  |  |
| **Toronto** |  |  | 171 |  |  | 172 |  |  | 172 |  |
|  | **CR** |  | 62 | -24.7 ± 1.6 |  | 62 | -24.2 ± 1.0 |  | 62 | -25.8 ± 1.4 |
|  |  | 0 | 18 | -24.6 ± 1.4 |  | 18 | -24.1 ± 1.1 |  | 18 | -25.0 ± 1.9 |
|  |  | 4 | 16 | -24.8 ± 1.7 |  | 16 | -24.2 ± 0.9 |  | 16 | -26.2 ± 0.9 |
|  |  | 8 | 15 | -24.8 ± 2.0 |  | 15 | -24.1 ± 1.1 |  | 15 | -25.9 ± 1.1 |
|  |  | 12 | 13 | -24.6 ± 1.4 |  | 13 | -24.5 ± 1.0 |  | 13 | -26.1 ± 1.0 |
|  |  |  |  |  |  |  |  |  |  |  |
|  | **D** |  | 45 | -24.9 ± 1.7 |  | 46 | -23.8 ± 1.0 |  | 46 | -26.0 ± 2.4 |
|  |  | 0 | 12 | -24.8 ± 2.0 |  | 13 | -23.9 ± 0.9 |  | 13 | -25.6 ± 1.4 |
|  |  | 4 | 10 | -24.7 ± 1.7 |  | 10 | -23.8 ± 1.1 |  | 10 | -26.9 ± 4.8 |
|  |  | 8 | 12 | -25.1 ± 1.5 |  | 12 | -24.0 ± 0.9 |  | 12 | -26.0 ± 1.0 |
|  |  | 12 | 11 | -25.0 ± 1.7 |  | 11 | -23.6 ± 1.0 |  | 11 | -25.7 ± 1.0 |
|  |  |  |  |  |  |  |  |  |  |  |
|  | **DCR** |  | 64 | -25.1 ± 1.9 |  | 64 | -24.2 ± 0.9 |  | 64 | -25.7 ± 1.3 |
|  |  | 0 | 17 | -26.0 ± 2.3 |  | 17 | -24.4 ± 1.1 |  | 17 | -25.6 ± 1.2 |
|  |  | 4 | 16 | -25.2 ± 1.9 |  | 16 | -24.3 ± 0.8 |  | 16 | -25.8 ± 1.5 |
|  |  | 8 | 15 | -24.6 ± 1.6 |  | 15 | -23.9 ± 0.8 |  | 15 | -25.5 ± 1.5 |
|  |  | 12 | 16 | -24.5 ± 1.4 |  | 16 | -24.0 ± 0.7 |  | 16 | -25.9 ± 0.7 |

**
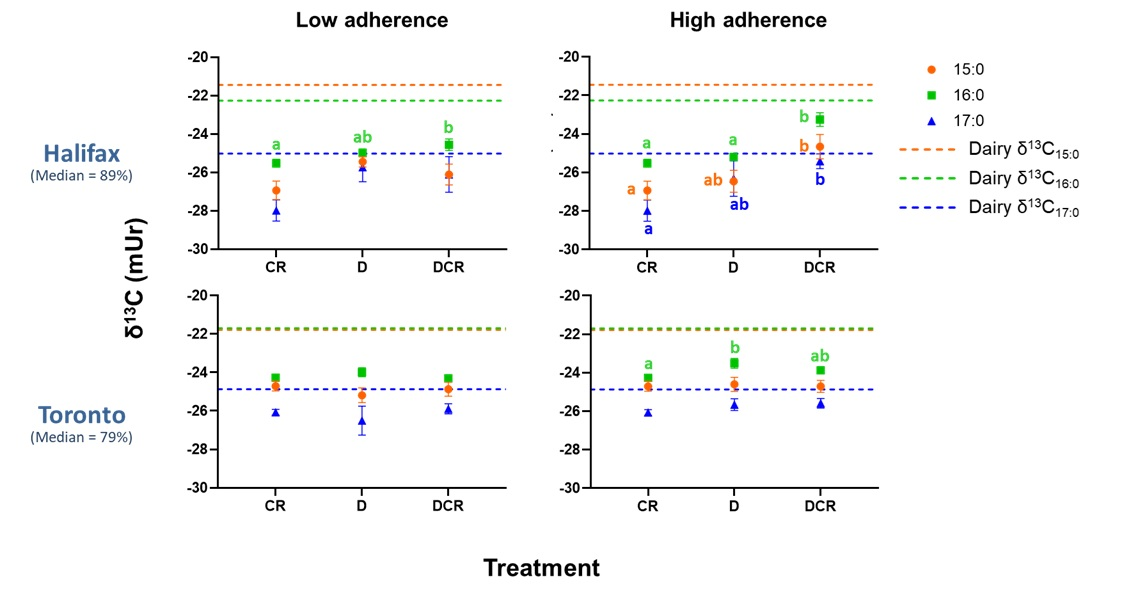
**

**Figure S15** Mean δ^13^C ratios of 15:0 (orange circles), 16:0 (green squares), and 17:0 (blue triangles) measured in the plasma of the participants in low- vs high-adherence groups of participants in Halifax and Toronto, across the calorie restricted (CR), dairy (D), and dairy-calorie restricted (DCR) treatments. Low- and high-adherence groups were established according to the relative median proportion of adherence of the participants to the assigned dairy treatments. Samples at weeks 4, 8, and 12 were grouped for each treatment. Error bars represent standard errors (n_H_Low_ = 15-28, n_H_High_ = 11-28, n_T_Low_ = 20-44, n_T_High_ = 12-44), while a letter code highlights significant (p < 0.05) Tukey’s pairwise comparisons across treatments following one-way analysis of variance (Table S16), and dashed lines identifies the δ^13^C ratios of 15:0 (orange), 16:0 (green), and 17:0 (blue) measured in the dairy items provided to the participants.

**Table S16** Results of one-way analysis of variance (ANOVA) tests performed to assess the differences across treatments in the δ^13^C ratios of plasma 15:0, 16:0, and 17:0 measured in low- vs high-adherence groups of participants in Halifax and Toronto. Low- and high-adherence groups were established according to the relative median proportion of adherence of the participants to the assigned dairy treatments (i.e., 89% for the Halifax participants, 79% for the Toronto participants).

| **Sampling Site** | **ANOVA table** | **SS** | **DF** | **MS** | **F (DFn, DFd)** | **P value** |
| --- | --- | --- | --- | --- | --- | --- |
| **Halifax-Low** |  |  |  |  |  |  |
|  | **δ^13^C_15:0_** |  |  |  |  |  |
|  | Treatment (between columns) | 21.870 | 2 | 10.930 | F (2, 55) = 2.314 | P=0.1084 |
|  | Residual (within columns) | 259.800 | 55 | 4.724 |  |  |
|  | Total | 281.700 | 57 |  |  |  |
|  |  |  |  |  |  |  |
|  | **δ^13^C_16:0_** |  |  |  |  |  |
|  | Treatment (between columns) | 10.180 | 2 | 5.092 | F (2, 57) = 4.472 | P=0.0157 |
|  | Residual (within columns) | 64.910 | 57 | 1.139 |  |  |
|  | Total | 75.090 | 59 |  |  |  |
|  |  |  |  |  |  |  |
|  | **δ^13^C_17:0_** |  |  |  |  |  |
|  | Treatment (between columns) | 54.120 | 2 | 27.060 | F (2, 48) = 3.079 | P=0.0552 |
|  | Residual (within columns) | 421.800 | 48 | 8.788 |  |  |
|  | Total | 475.900 | 50 |  |  |  |
| **Halifax-High** |  |  |  |  |  |  |
|  | **δ^13^C_15:0_** |  |  |  |  |  |
|  | Treatment (between columns) | 41.160 | 2 | 20.580 | F (2, 50) = 3.688 | P=0.0321 |
|  | Residual (within columns) | 279.000 | 50 | 5.580 |  |  |
|  | Total | 320.200 | 52 |  |  |  |
|  |  |  |  |  |  |  |
|  | **δ^13^C_16:0_** |  |  |  |  |  |
|  | Treatment (between columns) | 41.790 | 2 | 20.890 | F (2, 51) = 20.940 | P<0.0001 |
|  | Residual (within columns) | 50.880 | 51 | 0.998 |  |  |
|  | Total | 92.670 | 53 |  |  |  |
|  |  |  |  |  |  |  |
|  | **δ^13^C_17:0_** |  |  |  |  |  |
|  | Treatment (between columns) | 55.490 | 2 | 27.750 | F (2, 42) = 4.528 | P=0.0166 |
|  | Residual (within columns) | 257.400 | 42 | 6.128 |  |  |
|  | Total | 312.900 | 44 |  |  |  |
|  |  |  |  |  |  |  |
|  |  |  |  |  |  |  |
| **Toronto-Low** |  |  |  |  |  |  |
|  | **δ^13^C_15:0_** |  |  |  |  |  |
|  | Treatment (between columns) | 3.141 | 2 | 1.571 | F (2, 85) = 0.5341 | P=0.5881 |
|  | Residual (within columns) | 249.900 | 85 | 2.941 |  |  |
|  | Total | 253.100 | 87 |  |  |  |
|  |  |  |  |  |  |  |
|  | **δ^13^C_16:0_** |  |  |  |  |  |
|  | Treatment (between columns) | 1.364 | 2 | 0.682 | F (2, 85) = 0.6940 | P=0.5024 |
|  | Residual (within columns) | 83.510 | 85 | 0.983 |  |  |
|  | Total | 84.880 | 87 |  |  |  |
|  |  |  |  |  |  |  |
|  | **δ^13^C_17:0_** |  |  |  |  |  |
|  | Treatment (between columns) | 4.284 | 2 | 2.142 | F (2, 85) = 0.6122 | P=0.5445 |
|  | Residual (within columns) | 297.400 | 85 | 3.499 |  |  |
|  | Total | 301.700 | 87 |  |  |  |
| **Toronto-High** |  |  |  |  |  |  |
|  | **δ^13^C_15:0_** |  |  |  |  |  |
|  | Treatment (between columns) | 0.111 | 2 | 0.056 | F (2, 76) = 0.02266 | P=0.9776 |
|  | Residual (within columns) | 186.500 | 76 | 2.454 |  |  |
|  | Total | 186.600 | 78 |  |  |  |
|  |  |  |  |  |  |  |
|  | **δ^13^C_16:0_** |  |  |  |  |  |
|  | Treatment (between columns) | 6.200 | 2 | 3.100 | F (2, 76) = 3.733 | P=0.0284 |
|  | Residual (within columns) | 63.110 | 76 | 0.830 |  |  |
|  | Total | 69.310 | 78 |  |  |  |
|  |  |  |  |  |  |  |
|  | **δ^13^C_17:0_** |  |  |  |  |  |
|  | Treatment (between columns) | 4.187 | 2 | 2.093 | F (2, 76) = 1.802 | P=0.1720 |
|  | Residual (within columns) | 88.310 | 76 | 1.162 |  |  |
|  | Total | 92.490 | 78 |  |  |  |
